# The genetic basis of diurnal preference in *Drosophila melanogaster*

**DOI:** 10.1186/s12864-020-07020-z

**Published:** 2020-08-31

**Authors:** Mirko Pegoraro, Laura M. M. Flavell, Pamela Menegazzi, Perrine Colombi, Pauline Dao, Charlotte Helfrich-Förster, Eran Tauber

**Affiliations:** 1 Department of Genetics and Genome Biology, University of Leicester, University Road, Leicester, LE1 7RH UK; 2 School of Natural Science and Psychology, Liverpool John Moores University, Liverpool, L3 3AF UK; 3 Neurobiology and Genetics, Biocenter, University of Würzburg, Würzburg, Germany; 4 Department of Evolutionary and Environmental Biology and Institute of Evolution, University of Haifa, 3498838 Haifa, Israel

**Keywords:** Circadian clock, *Drosophila*, Diurnal preference, Nocturnality

## Abstract

**Background:**

Most animals restrict their activity to a specific part of the day, being diurnal, nocturnal or crepuscular. The genetic basis underlying diurnal preference is largely unknown. Under laboratory conditions, *Drosophila melanogaster* is crepuscular, showing a bi-modal activity profile. However, a survey of strains derived from wild populations indicated that high variability among individuals exists, including flies that are nocturnal.

**Results:**

Using a highly diverse population, we performed an artificial selection experiment, selecting flies with extreme diurnal or nocturnal preference. After 10 generations, we obtained highly diurnal and nocturnal strains. We used whole-genome expression analysis to identify differentially expressed genes in diurnal, nocturnal and crepuscular (control) flies. Other than one circadian clock gene (*pdp1*), most differentially expressed genes were associated with either clock output (*pdf, to*) or input (*Rh3*, *Rh2, msn*). This finding was congruent with behavioural experiments indicating that both light masking and the circadian pacemaker are involved in driving nocturnality.

**Conclusions:**

Our study demonstrates that genetic variation segregating in wild populations contributes to substantial variation in diurnal preference. We identified candidate genes associated with diurnality/nocturnality, while data emerging from our expression analysis and behavioural experiments suggest that both clock and clock-independent pathways are involved in shaping diurnal preference. The diurnal and nocturnal selection strains provide us with a unique opportunity to understand the genetic architecture of diurnal preference.

## Background

Although time is one of the most important dimensions that define the species ecological niche, it is often a neglected research area [1]. Most animal species exhibit locomotor activity that is restricted to a defined part of the day, and this preference constitutes the species-specific *temporal niche*. Selection for activity during a specific time of the day is driven by various factors, including preferred temperature or light intensity, food availability and predation. The genetic basis for such phase preference is largely unknown and is the focus of this study.

The fact that diurnality preference is usually similar within phylogenetic groups [2] alludes to an underlying genetic mechanism. The nocturnality of mammals, for example, was explained by the nocturnal bottleneck hypothesis [2], which suggests that all mammals descended from a nocturnal ancestor. Nocturnality and diurnality most likely evolved through different physiological and molecular adaptations [3]. Two plausible systems that have been targeted for genetic adaptations driven by diurnal preference are the visual system and the circadian clock, the endogenous pacemaker that drives daily rhythms. The visual system of most mammals is dominated by rods, yet lacks several cone photoreceptors that are present in other taxa where a nocturnal lifestyle is maintained [4].

Accumulating evidence suggests that diurnal preference within a species is far more diverse than previously thought. Laboratory studies [1] have often focused on a single representative wild-type strain and ignored the population and individual diversity within a species. In addition, experimental conditions in the laboratory setting (particularly light and temperature) often fail to simulate the high complexity that exists in natural conditions [1]. Furthermore, many species shift their phase preference upon changes in environmental conditions. Such “temporal niche switching” is undoubtedly associated with considerable plasticity that can lead to rapid changes in behaviour. For example, the spiny mouse (*Acomys cahirinus*) and the golden spiny mouse (*A. russatus*) are two sympatric desert species that split their habitat, with the common spiny mouse being nocturnal and the golden spiny mouse being diurnal. However, in experiments where the golden spiny mouse was the only species present, the mice immediately reverted to nocturnal behaviour [5].

While plasticity plays an important role in diurnal preference, there is evidence for a strong genetic component underlying the variability seen among individuals. For instance, twin studies [6] found higher correlation of diurnal preference in monozygotic twins than in dizygotic twins, with the estimated heritability being as high as 40%. In addition, a few studies in humans reported a significant association between polymorphisms in circadian clock genes and ‘morningness–eveningness’ chronotypes, including a polymorphism in the promoter region of the *period3* gene [7].

*Drosophila melanogaster* is considered a crepuscular species that exhibits a bimodal locomotor activity profile (in the laboratory), with peaks of activity appearing just before dawn and dusk. This pattern is highly plastic and the flies promptly respond to changes in day-length or temperatures simulating winter or summer. It has been shown that increases in temperature or irradiance during the day drive the flies to nocturnality, whereas low temperatures or irradiances result in a shift to more prominent diurnal behaviour [8, 9]. Such plasticity was also demonstrated in studies showing that flies switch to nocturnality under moonlight [10, 11] or in the presence of other socially interacting flies [12].

Available evidence also alludes to the genetic component of phase preference in *Drosophila*. Sequence divergence in the *period* gene underlies the phase difference seen in locomotor and sexual rhythms between *D. melanogaster* and *D. pseudoobscura* [13]. Flies also show natural variation in the timing of adult emergence (eclosion), with a robust response to artificial selection for the early and late eclosion phases having been shown, indicating that substantial genetic variation underlies this trait [14].

Further support for a genetic component to phase preference comes from our previous studies of allelic variation in the circadian-dedicated photoreceptor *cryptochrome* (CRY), where an association between a pervasive replacement SNP (L232H) and the phases of locomotor activity and eclosion was revealed [15]. Studies of null mutants of the *Clock* gene (*Clk*^*jrk*^) revealed that such flies became preferentially nocturnal [16], and that this phase switch is mediated by elevated CRY in a specific subset of clock neurons [17]*.* In other experiments, mis-expression of *Clk* resulted in light pulses evoking longer bouts of activity, suggesting that *Clk* plays a clock-independent role that modulates the effect of light on locomotion [18].

Here, using 272 natural population strains from 33 regions in Europe and Africa, we generated a highly diverse population whose progeny exhibited a broad range of phase preferences, with both diurnal and nocturnal flies being counted. We exploited this phenotypic variability to study the genetic architecture of diurnal preference and identify loci important for this trait using artificial selection, selecting for diurnal and nocturnal flies.

## Results

### Artificial selection for diurnal preference

**Fig. 1:**
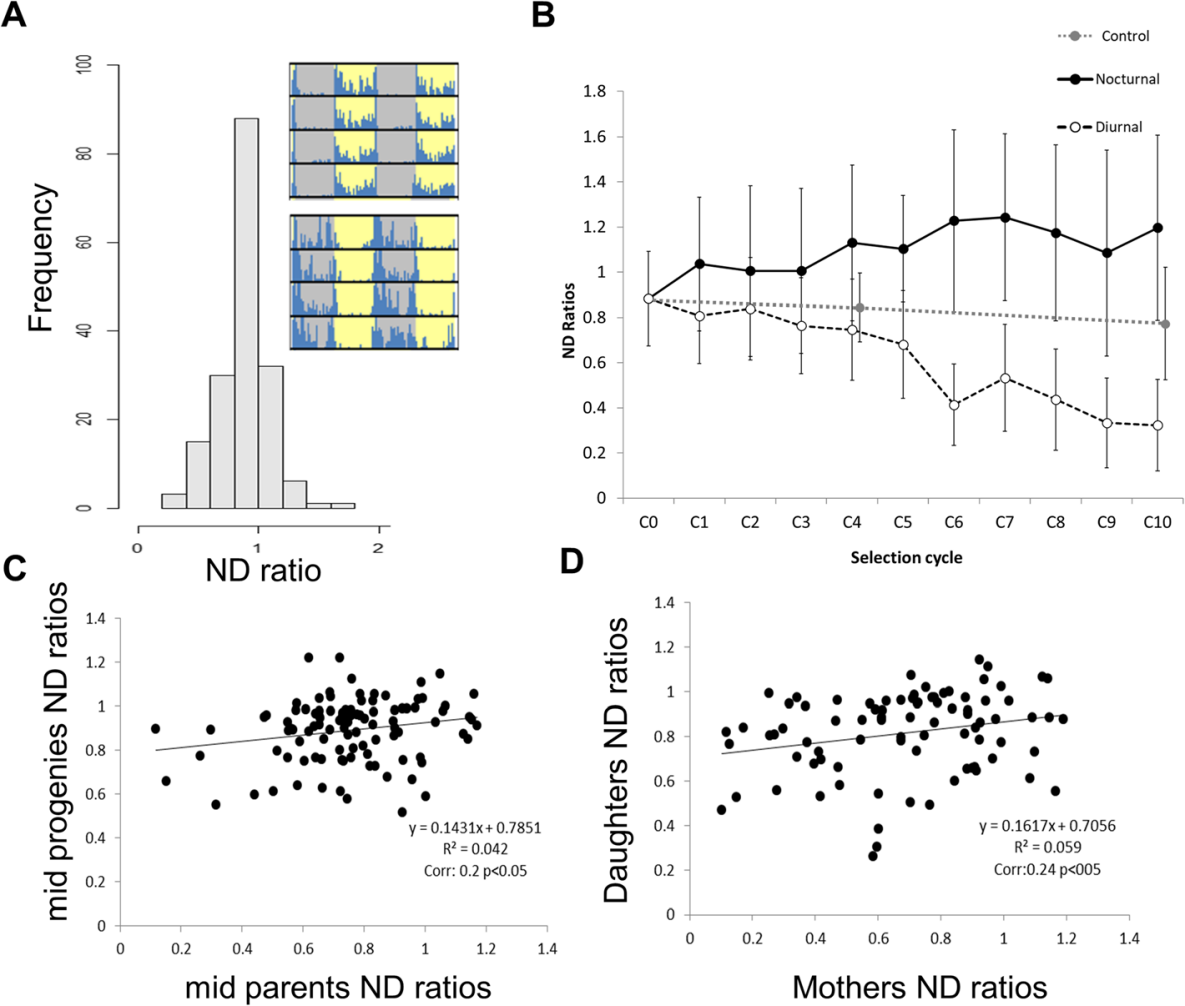
Responses to artificial selection of nocturnal/diurnal locomotor activity. **a.** Distribution of ND ratios of males from the starting population (C_0_, *n* = 176). The insets show actogram examples of diurnal (top) and nocturnal flies (bottom). Grey and yellow shading represent night and day, respectively. **b.** Average ND ratios of males from selected populations per cycle of selection. The black solid line is the nocturnal selection, while the dashed line is the diurnal selection. Data points correspond to average ND ratios ± standard deviation (*n* = 104–316). Grey points at the 4th and 10th cycle of selection correspond to ND ratios for the unselected control population (*n* = 19–78). The ND ratio of the original population is shown at C0. **c.** Correlation between mid-parent (*n* = 105) and mid-progeny (*n* = 105). Correlation coefficients and *p* values are reported below the regression eq. **d.** Correlation between mothers (*n* = 85) and daughters (*n* = 85)

Flies showed a rapid and robust response to selection for phase preference. After 10 selection cycles, we obtained highly diurnal (D) and nocturnal (N) strains. The two control strains (C) showed intermediate (crepuscular) behaviour (Fig. 1). To quantify diurnal preference, we defined the ND ratio, quantitatively comparing activity during a 12 h dark period and during a 12 h light period. As early as after 1 cycle of selection, the ND ratios of N and D flies were significantly different, relative to the original (control) population (Fig. 1a, b). After 10 generations of selection, the N and D populations were highly divergent (Fig. 1b, Table S1).

The estimated heritability h^2^ was higher for diurnality (37.1%) than for nocturnality, (8.4%) reflecting the asymmetric response of the two populations (Table S1). Using flies from the initial population (C0, see Material and methods), we also estimated heritability due to parent-offspring regression (Fig. 1c, d) and saw that narrow-sense heritability was lower but significant (Fig. 1c; h^2 =^ 14% *p* < 0.05). The heritability value was slightly higher when ND ratios of mothers and daughters were regressed (Fig. 1d; h^2 =^ 16% *p* < 0.05), although such values were minute and not significant in cases of father-son regression (Supplementary Figure S1, h^2 =^ 2.5% NS).

### Effects of nocturnal/diurnal phenotypes on fitness

**Fig. 2:**
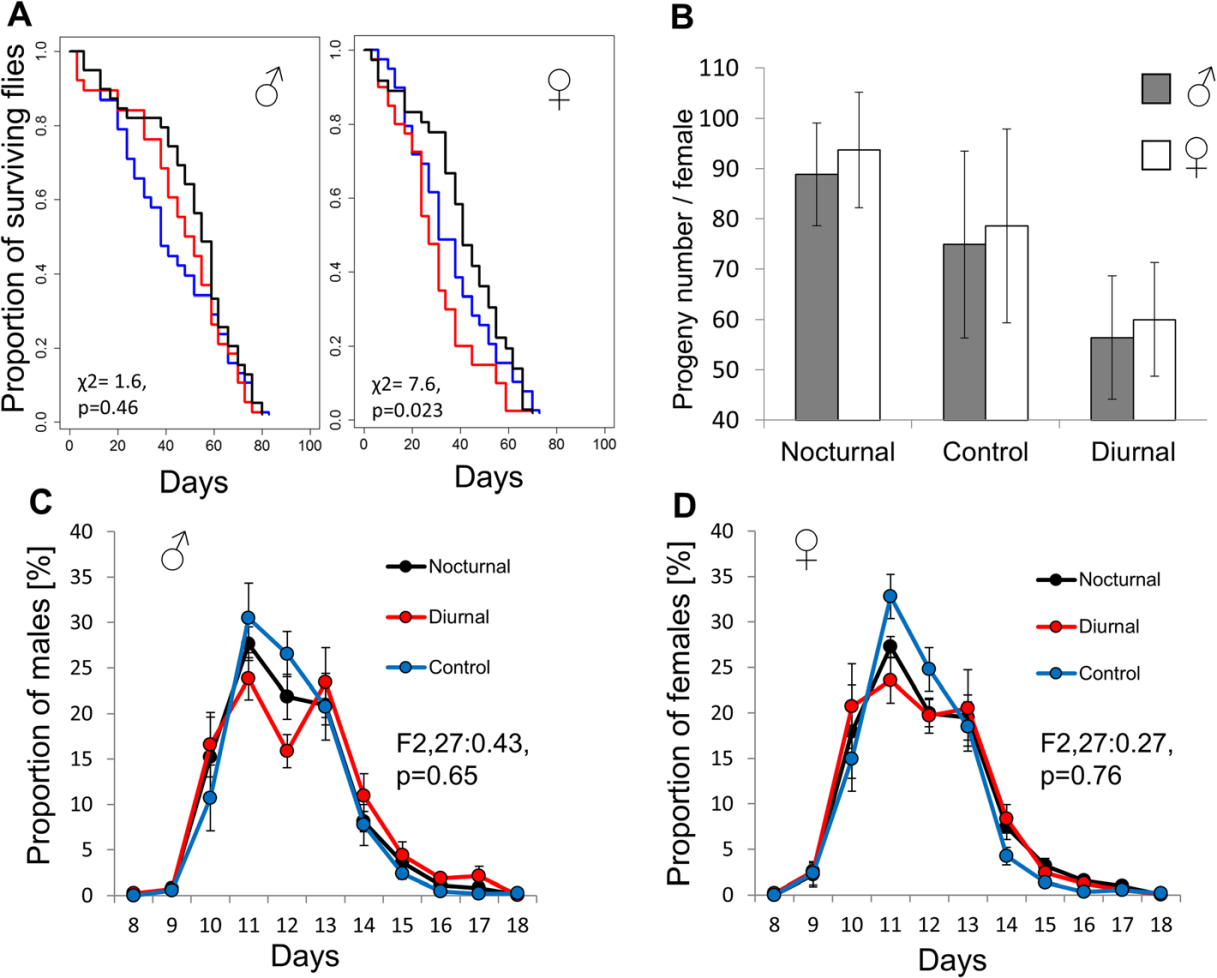
Correlated responses of fitness traits to selection. **a.** Survival curves of flies from the three selection populations (*n* = 36–40; N: black, D: red, C: blue). The proportion of surviving flies (y axis) is plotted against the number of days (x axis). **b.** Number of progeny produced per female for N, C and D crosses. Grey and white bars indicate the number of males and females, respectively (average ± standard error). Development time (egg-to-adult) distributions are shown for male progeny (**c**) and females (**d**). Proportion (%) of progeny produced per female per day. Males: N (*n* = 3556), D (*n* = 2308) and C (*n* = 2998). Females: N (*n* = 3748), D (*n* = 2401) and C (*n* = 3145)

A possible mechanism driving the observed asymmetric response to selection is unequal allele frequencies, whereby a slower response to selection is associated with increased fitness [19]. We, therefore, tested whether our selection protocol asymmetrically affected the fitness of the N and D populations. After ~ 15 overlapping generations from the end of the selection, in which selection has been relaxed, the ND ratios of N strain flies decreased from 1.2 (C10) to 0.99, and those of D strain flies increased from 0.32 to 0.53. There were still significant differences noted between N and both D and C strain flies, but no difference between C and D flies (Figure S2). At this stage, we tested viability, fitness and egg-to-adult developmental time of the selection and control populations. While the survivorship of males from the three populations was similar (χ2 = 1.6, df = 2, *p* = 0.46; Fig. 2a), intriguingly, we found significant differences in females. N females lived significantly longer than D females, while C females showed intermediate values (χ2 = 7.6, df = 2, *p* < 0.05; Fig. 2a). The progeny number of N females was larger than that of D females, with C females showing intermediate values (♂F_1,18_ = 5.12, p < 0.05; ♀F_1,18_ = 5.09, *p* < 0.05; Fig. 2b). Developmental time (egg-to-adult), another determinant of fitness, did not differ significantly between the nocturnal/diurnal populations (**♂**F_2,27_ = 0.43, *p* = 0.65, NS; **♀** F_2,27_ = 0.27, *p* = 0.76, NS; Fig. 2c, d).

### Effects on circadian behaviour

Since the circadian system is a conceivable target for genetic adaptations that underlie diurnal preference, we tested whether the circadian clock of the N and D strains were affected by the Nocturnal/Diurnal artificial selection. Accordingly, we recorded the locomotor activity of the selection lines following three generations after completion of the selection protocol, and measured various parameters of circadian rhythmicity.

The phase of activity peak in the morning (MP) and in the evening (EP) differed between the populations (Figure S3A). As expected, the MP of the N population was significantly advanced, as compared to that seen in both the C and D populations. The EP of the N population was significantly delayed, as compared to that noted in the two other populations. Concomitantly, sleep pattern was also altered (Figure S3B), with N flies sleeping much more during the day than did the other populations. While D flies slept significantly more than C and N flies during the night, there was no difference in the amount of sleep between N and C flies.

**Fig. 3:**
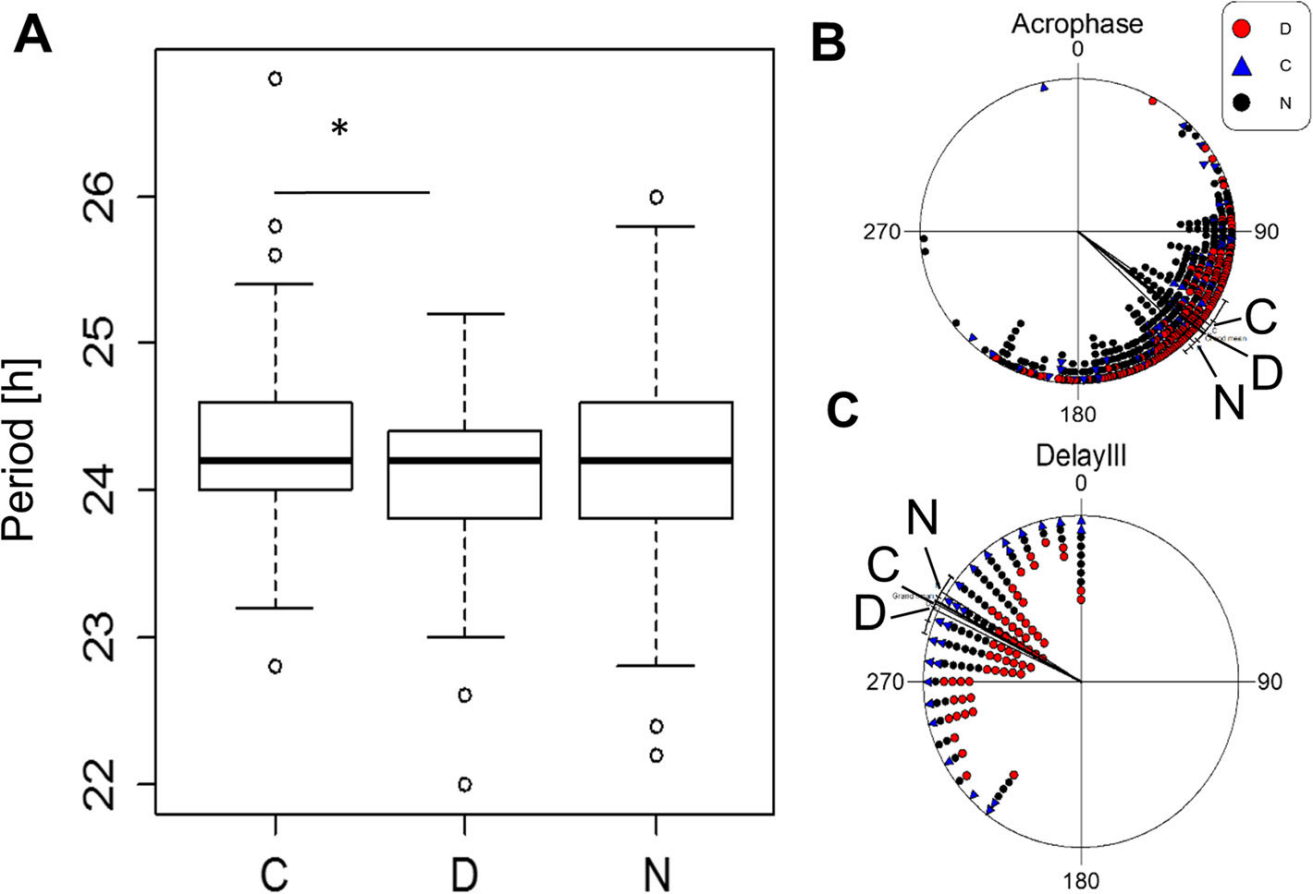
Circadian behaviour of nocturnal and diurnal selection flies. **a.** Boxplots of circadian periods under free-run conditions. Statistical differences were tested by a TukeyHSD test, with * signifying *p* < 0.05. **b.** Acrophase angles of the free-run activity for N (black circles, *n* = 255), D (red circles, *n* = 159) and C (blue triangles, *n* = 65) populations. The free-run phases of the three populations did not differ (F_2,476_:1.91, *p* = 0.149, NS). Lines represent mean vectors ±95% confidence interval (CI). One hour corresponds to an angle of 15°. **C**. Phase delay angles are shown for N (black circles, *n* = 153), D (red circles, *n* = 155) and C (blue triangles, *n* = 56) populations. Differences are not significant (F_2,361_ = 1.47, *p* = 0.23, NS). Lines represent mean vectors ±95% CI

In contrast to the striking differences seen between the selection lines under light-dark (LD) conditions, smaller differences were observed under continuous darkness (DD) conditions (Fig. 3, Figure S3C). The period of free-run of activity (FRP) was longer in C flies than in D flies, while the difference between the D and N groups was only marginally significant (Fig. 3a). No significant difference in FRP was found between N and C flies. The phases (φ) of the three populations did not differ significantly (Fig. 3b). We also tested the responses of the flies to an early night (ZT15) light stimulus and found no significant differences in their delay responses (Fig. 3c).

### Circadian differences between isogenic nocturnal/diurnal strains

To facilitate genetic dissection of nocturnal/diurnal preference, we generated isogenic nocturnal, diurnal and control strains (D*, N* and C*; one of each) from the selected populations. The strains were generated using a crossing scheme involving strains carrying balancer chromosomes. The ND ratios of the isogenic lines resembled those of the progenitor selection lines (Figure S4A). The isogenic strains also differed in terms of their sleep pattern (Figure S4B).

The circadian behaviour of the isogenic lines differed, with the N* line having a longer FRP than both the D* and C* lines (Figure S5A). The locomotory acrophase of the N* line was delayed by about 2 h, as compared to the D* line, and by 1.38 h, as compared to the C* line (F_2,342_ = 6.01, *p* < 0.01; Figure S5B). In contrast, circadian photosensitivity seemed to be similar among the lines, as their phase responses to a light pulse at ZT15 did not differ (F_2,359_:1.93, *p* = 0.15, NS; Figure S5D). Since eclosion is regulated by the circadian clock [20, 21], we also compared the eclosion phase of the isogenic strains. Under LD, the eclosion phase of D* flies was delayed by ~ 2 h (becoming more diurnal), as compared to both N* and C* flies, whereas no difference between N* and C* flies was detected (Figure S5C).

### Diurnal preference is partly driven by masking

**Fig. 4:**
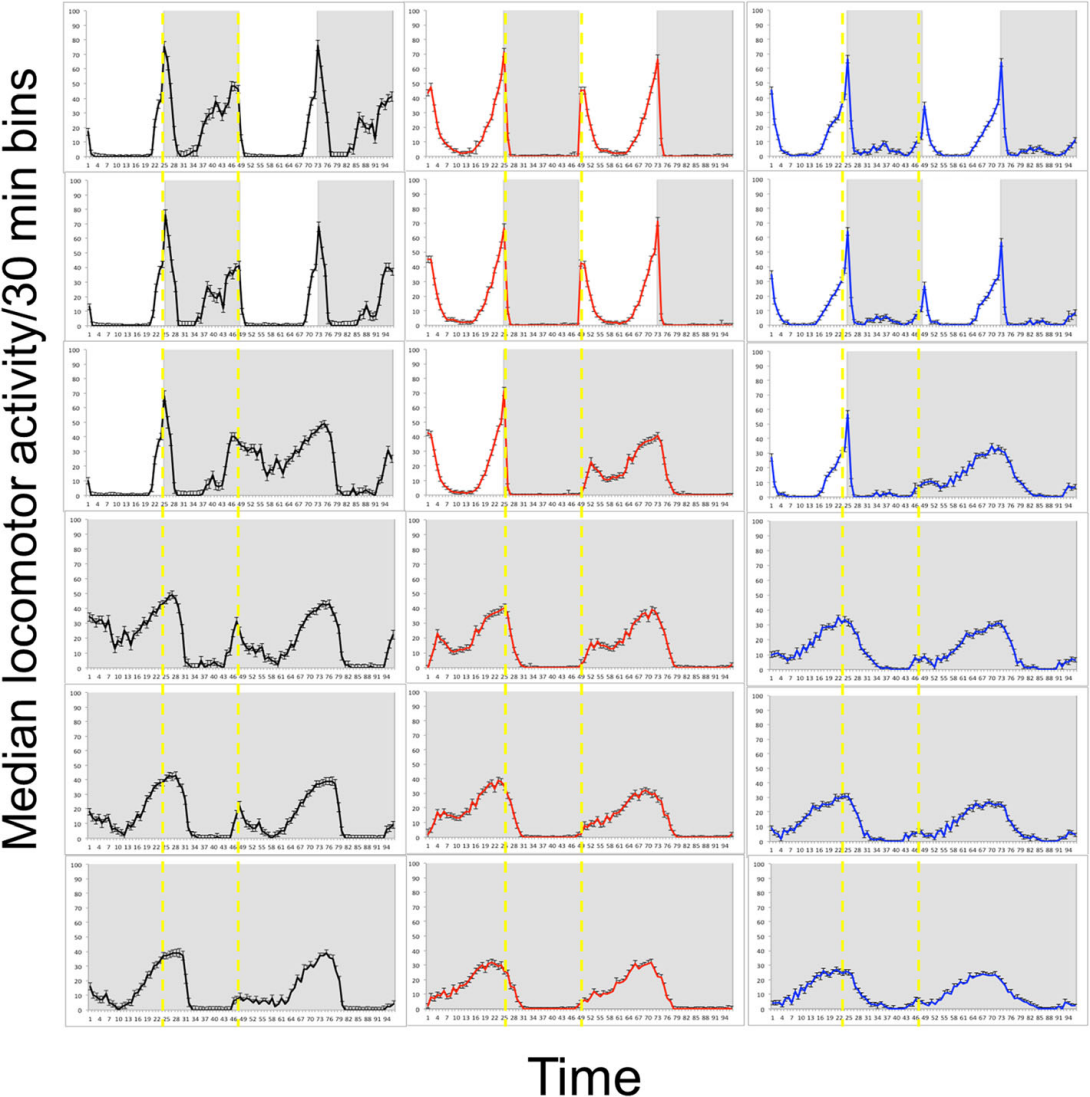
Light masking of locomotor behaviour. Double plots of median locomotor activity (± SEM) per 30 min bin during 3 days in a 12:12 LD cycle followed by 4 days in DD. (N*, black, *n* = 71; D*, red, *n* = 130; C*, Blue, *n* = 110). Shades indicate light-off. Yellow dashed lines delineate subjective nights. The overall ANOVA between 4 days in LD and 4 days in DD conditions (starting from the second day in DD) indicates a significant effect of the light regime (i.e., LD vs. DD; F_1,594_ = 105.03, *p* < 0.0001) and genotype (F_2,594_:173.01, *p* < 0.0001). The interaction genotype × environment was also significant (F_2,594_:102.66, *p* < 0.0001). Post-hoc analysis (TukeyHSD text) revealed that both N* and C* flies became significantly more “diurnal” when released into constant conditions (N* *p* < 0.0001; C* *p* < 0.001). The ND ratios of D* flies did not significantly change in DD conditions (*p* = 0.94, NS)

We reasoned that light masking (i.e., the clock-independent inhibitory or stimulatory effect of light on behaviour) could be instrumental in driving diurnal preference. We thus monitored fly behaviour in DD conditions to assess the impact of light masking. We noticed that when N* flies were released in DD conditions, their nocturnal activity was much reduced, whereas their activity during the subjective day increased (Fig. 4). Indeed, the behaviour of N* and D* flies in DD conditions became quite similar (Fig. 4). Congruently, when we analysed the ND ratios of these flies in LD and DD conditions, we found that that both N* and C* flies became significantly more “diurnal” when released into constant conditions (N*, *p* < 0.0001; C*, *p* < 0.001). In contrast, the ratios of D* flies did not significantly change in DD conditions (*p* = 0.94, NS). This result suggests that nocturnal behaviour is at least partially driven by a light-dependent repression of activity (i.e., a light masking effect).

### Correlates of the molecular clock

**Fig. 5:**
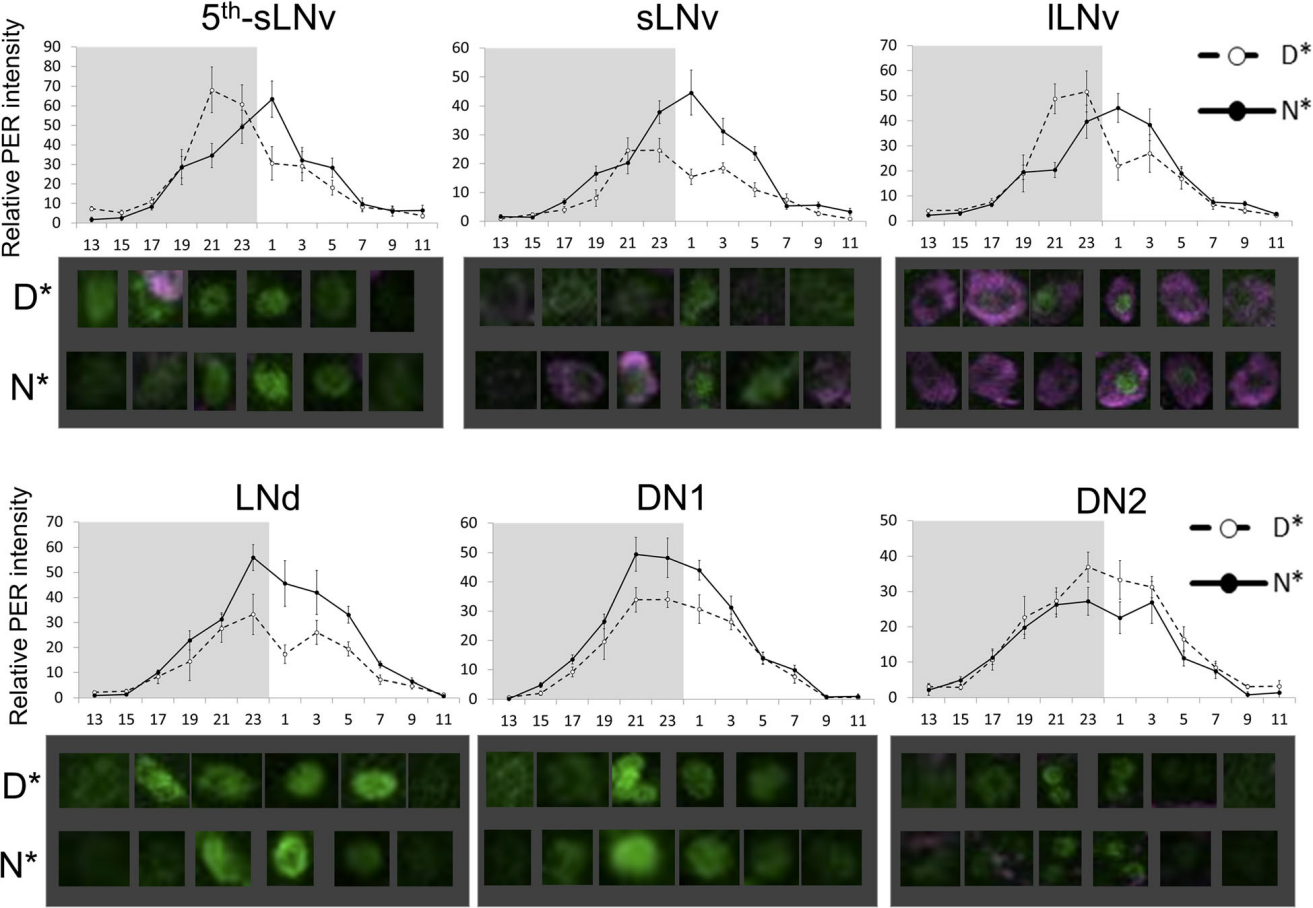
Expression of PER in clock neurons. Quantification of nuclear PER staining in N* (full lines) and D*(dashed lines) flies maintained in LD conditions. Shaded area represents dark. Representative staining (composite, Z-stacks) is shown below each panel (ZT13-ZT9, 4 h intervals). Points represent averages ± standard error. The appearance of the peak PER signal in ventral neurons (LNv: 5th-sLNv, sLNv, lLNv) was delayed in N* flies, as compared to D* flies (ZT1 vs ZT21–23), as indicated by significant time x genotype interactions: 5th-sLNv χ2 = 12,141, df = 11, *p* < 0.0001; sLNv χ2 = 4779.4, df = 11, *p* < 0.0001; lLNv χ2 = 7858, df = 11, *p* < 0.0001. The N* PER signal is stronger in sLNv (χ2 = 2416.6, df = 1, *p* < 0.0001), LNd (χ2 = 3924, df = 1, *p* < 0.0001) and DN1 (χ2 = 1799, df = 1, *p* < 0.0001) and weaker in DN2 (χ2 = 523.2, df = 1, *p* < 0.05)

To investigate whether differences in diurnal preference correlated with a similar shift in the molecular clock, we measured the intensity of nuclear PERIOD (PER) in key clock neurons (Fig. 5). The peak of PER signals in ventral neurons (LNv: 5th-sLNv, sLNv, lLNv) was delayed in N* flies, as compared to the timing of such signals in D* fly 5th-sLNv, sLNv and lLNv neurons. In N* and D* flies, the phases of such peaks in dorsal neurons (DN, including the clusters LNd, DN1and DN2) were similar (Fig. 5).

We also measured the expression of the Pigment Dispersing Factor (PDF) in LNv projections (Figure S6-S7). The signal measured in N* flies was lower than that measured in D* flies during the first part of the day (in particular at ZT3 and ZT7), yet increased during day-night transition at ZT11 and ZT13. There were no differences seen during the rest of the night.

### Global transcriptional differences between nocturnal/diurnal strains

To gain insight into the genetics of diurnal preference, we profiled gene expression in fly heads of individuals from the D*, C* and N* isogenic lines by RNAseq. We tested for differentially expressed genes (DEG) in all pairwise contrasts among the three strains at two time points. We found 34 DEGs at both ZT0 and ZT12 (Table S3). An additional 19 DEG were unique to ZT0 and 87 DEG were unique to ZT12 (Table S3). Functional annotation analysis (DAVID, https://david.ncifcrf.gov/ [22]) revealed similarly enriched categories at ZT0 and ZT12 (Figure S8). The predicted products of the DEGs were largely assigned to extracellular regions and presented secretory pathway signal peptides. DEG products identified only at ZT12 were related to the immune response, amidation and kinase activity. Given the intermediate phenotype exhibited by C* flies, we reanalysed the data, searching for DEGs where C* flies showed intermediate expression (D* > C* > N* or N* > C* > D*; Table S4). The list of DEGs consisted of 22 genes at ZT0 and 62 at ZT12. Amongst the different functions represented by these new lists were photoreception, circadian rhythm, and sleep, Oxidation-reduction and mating behaviour were over-represented in both D* and N* flies. For example, *Rhodopsin 3* (*Rh3*) was up-regulated in D* flies, while *Rh2* and *Photoreceptor dehydrogenase* (*Pdh*) were down-regulated in N* flies. *Pastrel* (*pst*), a gene involved in learning and memory, was up-regulated in D* flies, while genes involved in the immune response were up-regulated in N* flies. The only core clock gene that showed differential expression was *Par Domain Protein 1* (*Pdp1*), which was up-regulated in D* flies. The clock output genes *takeout* (*to*) and *pdf* were up-regulated in N* flies. Overall, the results suggest that genes that are transcriptionally associated with diurnal preference are mostly found upstream (light input pathways), and downstream of genes comprising the circadian clock.

### Complementation test

**Fig. 6:**
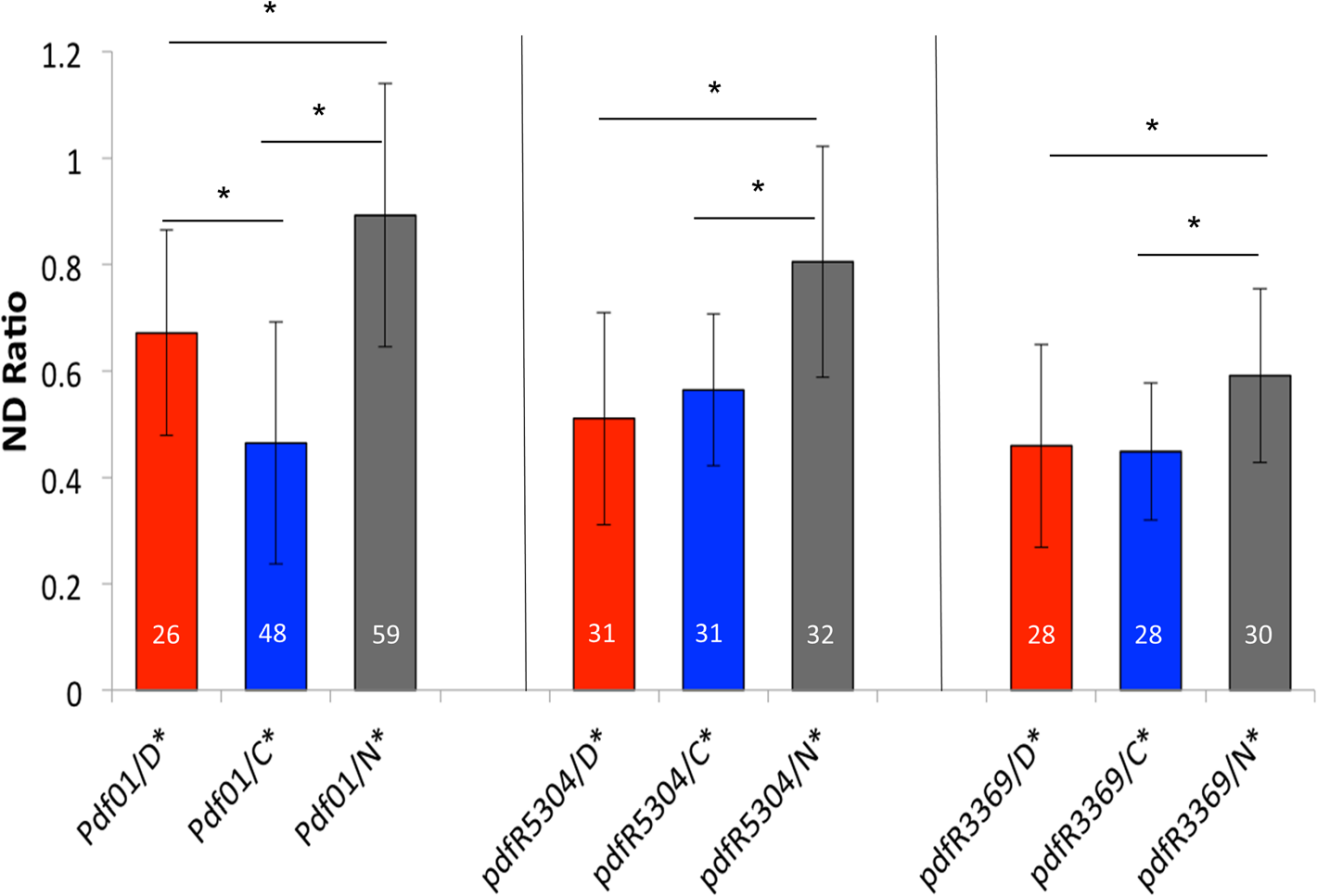
Quantitative complementation tests. Tests were performed to determine whether N*, D* and C* alleles vary in terms of their ability to complement the phenotype caused by the *Pdf* and *Pdfr* mutant alleles. Average ND ratio ± standard deviation of *Pdf*^*01*^ heterozygotes (left) and of *Pdfr* using p-element insertions *Pdfr*^*5304*^ (middle) and *Pdfr*^*3369*^ (right) are shown. Numbers of tested flies are reported in each chart bar. * represents *p* < 0.05

We investigated the contribution of various genes to nocturnal/diurnal behaviour using a modified version of the quantitative complementation test (QCT) [23]. Briefly, each of the natural alleles of a candidate gene is tested in association with a mutant allele of that gene, and their phenotypes are compared. We accordingly tested the core circadian clock genes *per* and *Clk*, the circadian photoreceptor *cry* and the output gene *Pdf* and *Pdfr*, encoding the Pdf receptor (Fig. 6, Table S5) [24]. We also tested the ion channel-encoding *narrow abdomen* (*na*) gene, given its role in the circadian response to light and dark-light transition [25]. QCT revealed significant allele differences in *per*, *Pdf*, *Pdfr*, *cry* and *na* (Fig. 6, Table S5), indicative of genetic variability in these genes contributing to the nocturnal/diurnal behaviour of the isogenic lines.

Since switching from nocturnal to diurnal behaviour in mice has been shown to be associated with metabolic regulation [26], we also tested *Insulin-like peptide 6* (*Ilp6*), and *chico*, both of which are involved in the *Drosophila* insulin pathway. A significant effect was found in *Ilp6* but not in *chico* (Table S5). Other genes that failed to complement were *paralytic* (*para*), encoding a sodium channel, and *coracle* (*cora*), involved in embryonic morphogenesis [27, 28].

We also tested genes that could affect the light input pathway, such as *Arrestin2* (*Arr2*) and *misshapen* (*msn*) [29]. Significant evidence was noted for *msn* failing to complement but not for *Arr2* (Table S5). Various biological processes are associated with *msn*, including glucose metabolism, as suggested by a recent study [30].

## Discussion

In this manuscript we addressed the genetic architecture that causes animals to be diurnal or nocturnal. Despite the vast importance of this trait in animal ecological niches, little is known about the genetic architecture underlying this behaviour. While diurnal preference is often stereotypic of a species [1], in some cases, including Mongolian gerbils [31], goldfish [32], carpenter ants [33], cotton rats [34] and the Chilean degus [31], both diurnal and nocturnal individuals are present. Our finding of such polymorphic diurnal preference in *Drosophila* paves the way for genetic dissection of this behaviour.

Here, we used artificial selection to generate two highly divergent populations that respectively showed diurnal and nocturnal activity profiles. The response to selection was asymmetrical, as reflected by heritability h^2^, which was higher for diurnality (37.1%) than for nocturnality (8.4%). This may indicate that different alleles and/or different genes were affected in the two nocturnal/diurnal selections. Selections for traits affecting fitness have been shown to have higher selection responses in the direction of lower fitness [19]. This may reflect the original (natural) allele frequency, whereby deleterious traits are mostly represented by recessive alleles and favourable traits are represented by alleles at high frequencies [19]. This asymmetrical allele frequency could generate non-linear heritability, such that a slower response to selection (as seen with nocturnal flies) is associated with increased fitness [19]. Indeed, nocturnal females lived longer and produced more progeny than did diurnal females, an observation that supports a scenario of asymmetric nocturnal/diurnal allele frequencies.

To what extent is the circadian clock involved in diurnal preference? We observed that (i) PER cycling in the lateral neuron was significantly shifted in nocturnal flies, and (ii) the phase of M and E peaks in DD differed between the strains, as did their free-running period (particularly in the isogenic strains). On the other hand, our data indicate that a non-circadian direct effect of light (light masking) played a significant role in diurnal preference, particularly nocturnality, as nocturnal flies in DD conditions become rather diurnal (Fig. 5). In rodents, the differential sensitivity of nocturnal and diurnal animals to light masking has been well documented [35]. This phenomenon was observed both in the laboratory and in the field [36], with light decreasing arousal in nocturnal animals and the opposite effect occurring in diurnal animals. Light masking in flies appears to have a greater impact, as it drives flies to nocturnality.

Notably, a comparison of gene expression between diurnal and nocturnal flies highlighted just a single core clock gene *(pdp1*). This finding is reminiscent of the results of our previous study in flies [37], where transcriptional differences between early and late chronotypes were present in genes up- and downstream of the clock but not in the clock itself. The phase conservation of core clock genes in diurnal and nocturnal animals has also been documented in mammals [38–40]. We thus suggest that selection for diurnal preference mainly targets downstream genes, thereby allowing for phase changes in specific pathways, as changes in core clock genes would have led to an overall phase change.

The main candidates responsible for diurnal preference that emerged from the current study were output genes, such as *pdf* (and the gene encoding the associated receptor *Pdfr*) and *to,* as well as genes involved in photoreception, such as *Rh3*, *TotA*, *TotC* (up-regulated in D* flies) and *Rh2* and *Pdh* (up-regulated in N*). Genes such as *misshapen (msn*) and *cry* were implicated by complementation tests. RH3 absorbs UV light (*λ*max = 347 nm) and is the rhodopsin expressed in rhabdomer 7 (R7) flies [41], while RH2 (*λ*max = 420 nm) is characteristic of the ocelli [42] and *pdh* is involved in chromophore metabolism [43].

Unlike diurnal/nocturnal preference, which is a rather binary trait, chronotype, another phase phenotype, is continuous in nature. The genetics of chronotype has been studied in various model systems, including humans (reviewed in ref. [44]). Twin and family studies have suggested that the heritability of chronotype is substantial, ranging from 14 to 50%, thus alluding to a significant genetic component. While earlier studies focused on candidate circadian clock genes, such as *per3* [45], recent GWAS studies allowed for unbiased identification of loci associated with chronotype variability in humans [46–48]. Five genes that showed significant association with chronotype were flagged by all three studies, with only two of these genes being linked to clock function (*per2* and *Rgs16*). Most of the associations that were identified in each of the studies did not serve a clear circadian function, again underscoring the premise that diurnal preference is regulated by multiple loci both within and outside the circadian clock.

## Conclusions

In this manuscript, we addressed the genetic architecture that causes animals to be diurnal or nocturnal. Despite the vast importance of this trait in animal ecological niches, little is known about the genes associated with this behaviour. A key finding of our study was that wild populations of *Drosophila* can exhibit extreme phenotypes, such as nocturnality or diurnality.

Using a highly diverse population, we performed an artificial selection experiment, selecting flies with extreme diurnal or nocturnal preference to obtain highly diurnal and nocturnal strains. We further used whole-genome expression analysis to identify differentially expressed genes among diurnal, nocturnal and crepuscular (control) flies. The transcriptional differences between nocturnal and diurnal flies that we identified are likely to be mediated by genetic variations in these genes or their transcriptional regulators. Our current effort is to identify those genetic variations which underlie the genetics of temporal niche preference. For this, the nocturnal and diurnal selection strains generated here will be an indispensable resource.

## Methods

The Supplemental Information contains extended experimental details (see Supplemental Methods).

### Artificial selection

To generate a highly genetically variable *Drosophila melanogaster* population, we pooled 5 fertilized females (4–5 days old) from 272 isofemale lines from 33 regions in Europe and Africa (Table S2) in the same culture bottle containing standard sugar food. This population was maintained at 25 °C in a 12:12 LD cycle. The progeny of this population was used in the artificial selection as generation 0 (C_0_; Fig. 1). The locomotor activity of 300 males was recorded over 5 days in a 12:12 LD cycle at 25 °C. Using the R library GeneCycle and a custom-made script, we identified rhythmic flies and calculated their ND ratios. In each cycle of selection, we selected 25 males with the most extreme nocturnal or diurnal ND ratios, and crossed them with their (unselected) virgin sisters.

### RNAseq

Gene expression was measured in head samples of flies collected at light-on (ZT0) and light-off (ZT12) times. Flies (3–4 days old) were trained for 3 days in a 12:12 LD cycle at 25 °C, after which male heads were collected in liquid nitrogen at ZT0 or ZT12. RNA was extracted using a Maxwell 16 MDx Research Instrument (Promega), combined with the Maxwell 16 Tissue Total RNA purification kit (AS1220, Promega). RNAseq library preparation and sequencing was carried by Glasgow Polyomics using an IlluminaNextseq500 platform.

## Supplementary information

**Additional file 1: Figure S1. Correlation between ND ratios of fathers and sons (*n*** **= 26 pairs)**. **Figure S2**. **Selection population ND ratios after 5 months of selection relaxation**. Distribution of ND ratios of males from the diurnal (D), nocturnal (N) and control (C) populations after 5 months (~ 15 generation) of selection relaxation. Average ND ± stdev values are reported for each line. The ND ratio of the N population was significantly different from both that of the control and D populations (Kolmogorov-Smirnov (KS) t-test for N vs C D = 0.56, *p* < 0.001, N vs D D = 0.68, *p* < 0.001). The ND ratios of the D and C populations were not significantly different (KS, D = 0.23, *p* = NS). **Figure S3. Locomotor behaviour and sleep of the selection lines in LD conditions**. **A.** LD acrophase angles of morning (MP) and evening (EP) peaks of activity for N (black circles, *n* = 230), D (red circles, *n* = 160) and C (blue triangles, *n* = 57) populations. Lines represent mean vectors±95%CI. One hour corresponds to a 15° angle. ZT0 and ZT12 are represented by 0° and 180° angles, respectively. The MP of N flies (*n* = 230) was significantly advanced, compared to that of both C (*n* = 57) and D (*n* = 160) flies, as tested by ANOVA (F_2,444_:163.87, *p* < 0.0001). The EP of N flies (*n* = 264) was significantly delayed, as compared to C (*n* = 82) and D (*n* = 170) flies; (F_2,513_:73.77, *p* < 0.0001). **B.** Total sleep, bins per hour shown for N (black, *n* = 304), D (red, *n* = 171) and C (blue, *n* = 100) populations. Data points correspond to averages ± SEM. The white/grey boxes represent day/night, respectively. N flies slept more during the day (*n* = 297) than did D (*n* = 166) and C (*n* = 96) flies (F_11,2224_ = 71.02, *p* < 0.0001). D flies slept more that did C and N flies during the night (F_11,2232_:93.45, *p* < 0.0001). There is no significant difference between N and C (TuskeyHSD, *p* = 0.08, NS) flies in terms of night sleep. **C.** The median locomotor activity per 30 min bin (±SEM) is shows for D (red), N (black) and C (blue) flies during the last day in a LD 12:12 cycle and the first 5 days in constant conditions (DD conditions, 25 °C). For each profile, the number of flies is depicted. **Figure S4. ND ratios and sleep of isogenic strains**. **A.** Distribution of ND ratios for males of the diurnal (D*), nocturnal (N*) and control (C*) isogenic lines. Average ND ± SD is reported for each line (note different X-axis scale). **B.** Total sleep bins per hour over time for N* (black, *n* = 70), D* (red, *n* = 170) and the C* (blue, *n* = 130) isogenic lines. Data points reflect averages ± SEM. The white/grey boxes represent day/night, respectively. Figure S5. Circadian behaviour of isogenic strains (N*, D* and C*). A. Boxplots of free-running periods of the N* (*n* = 64), C* (*n* = 124) and D* (*n* = 157) isogenic lines. Solid lines represent median periods, the bottom and upper ends of the box correspond to the upper and lower quartiles, respectively and the whiskers denote maximum and minimum values, excluding outliers. TukeyHSD test, *p < 0.05, **p < 0.001 **B.** Acrophase angles of the free-running activity shown for the N* (black circles, *n* = 64), D* (red circles, *n* = 157) and C* (blue triangles, *n* = 124) isogenic lines. The phase in the N* line was delayed by 2.02 h, as compared to what was measured in the D* line, and by 1.38 h, as compared to what was measured in the C* line (F_2,342_:6.01, *p* < 0.01). Lines represent mean vectors ±95% CI. One hour corresponds to a 15° angle. **C.** Phase of eclosion in the N* (black circle, *n* = 75), D* (red circle, *n* = 111) and C* (blue triangle, *n* = 50) lines. The eclosion phase of D* flies was delayed by ~ 2 h, as compared to what was measured with both the N* and C* lines (F_2,233_:4.95, *p* < 0.01). There was no difference between N* and C* flies (F_1,123_:0.08, *p* = 0.78, NS). Lines represent mean vectors ±95% CI. One hour corresponds to an angle of 15°. Light-on (ZT0) and light-off (ZT12) translated to 0° and 180° angles, respectively. **D.** Phase delays of N* (black circles, *n* = 66), D* (red circles, *n* = 170) and C* (blue triangles, *n* = 126) flies. There were no differences among the strains (F_2,359_:1.93, *p* = 0.15, NS). Lines represent mean vectors ±95% CI. **Figure S**6**. Representative PDF staining in LNv projections.** Representative PDF staining for LNv projections in N* (top) and D*(bottom) lines at ZT13 in flies maintained in a LD12:12 cycle at 25 °C. **Figure S7. Expression of PDF in LNv projections.** PDF staining in the N* (full lines) and D* (dashed lines) lines maintained in a 12:12 LD cycle. Shading represents light-off. Representative staining is shown in Supplementary Figure S4. Points represent averages ± standard error. The N* signal was lower than the D* signal at ZT3 (F_1,18_ = 11.99, *p* < 0.01) and ZT7 (F_1,19_ = 10.13, *p* < 0.01), and higher at ZT11 (F_1,15_ = 10.53, *p* < 0.01) and ZT13 (F_1,17_ = 23.39, *p* < 0.001). **Figure S8. Functional annotation of DEGs associated with diurnal preference.** Pie charts representing significant terms of DEGs in 3 pairwise contrasts (D* vs N*, D* vs C*, N* vs C*) at ZT0 and ZT12. Sections represent the percent of enrichment for each term. *p* < 0.05 after Benjamini correction, with the exception of the “signal peptide” term at ZT0, where *p* = 0.054. **Table S1. Artificial selection ND ratios and heritability**. ND ratios per cycle of selection (Cyc). Stdev indicates standard deviation and N is the number of rhythmic males. R is the response to selection and S is the selection differential per cycle of selection. Vp is variance of the ND ratio per cycle of selection and Var(h^2^) is the variance in h^2^ due to genetic drift. Cum R and Cum S correspond to the cumulative response to selection and the cumulative selection differential, respectively. KS D and pval are the results of the Kolmogorov-Smirnov test comparing ND ratios of two consecutive generations (*n* = 25 in all cases). **Table S2.** List of isofemale strains that were used for the synthetic population. **Table S3** List of differentially expressed genes (*p* < 0.05) in all pairwise contrasts. A group of 34 genes were differentially expressed both at ZT0 and ZT12 (**ZT0 ZT12**). Nineteen genes were uniquely identified at ZT0 (**ZT0)** and 87 were differentially expressed only at ZT12 (**ZT12**). Unknown transcripts are indicated by the suffix TCONS. **Table S4** Differentially expressed genes (*p* < 0.05). The biological processes as reported in flybase (http://flybase.org/). **Table S5** Complementation tests. Average ND ratios and standard deviations for each complementation test cross are shown. “n” indicates the number of tested flies. Kolmogorov-Smirnov tests (KS-test) are reported as matrixes with *p* values in the top half and D values in bottom half [49, 50]. Kruskal-Wallis rank sum test (KW-test) results with 2 degree of freedom are also shown for each complementation test [49, 50]. Compl indicates the result of the complementation test. Positive complementation was only confirmed for the ND D*cross<C*cross<N*cross, where the KS-test was significant for the N*cross vs. D*cross contrast; the KW-test was also significant. **Supplemental Methods**.

## Data Availability

The RNASeq sequencing files are available at the Gene Expression Omnibus (GEO) accession GSE116985. The Drosophila reference transcriptome (build 5.41) was obtained through Illumina iGenomes (https://emea.support.illumina.com/sequencing/sequencing_software/igenome.html).

## Abbreviations

D: Diurnal
N: Nocturnal
C: Control
MP: Morning peak
EP: Evening peak
LD: Light-dark
DD: Continuous darkness
FRP: Free-run activity
ZT: Zeitgeber time
