# Supplementary material for: The genetic basis of diurnal preference in Drosophila melanogaster
Source: BMC Genomics. 2020 Aug 31;21:596. doi: 10.1186/s12864-020-07020-z (PMC7457780; doi:10.1186/s12864-020-07020-z)
Supplement: Supplementary file 1 — Additional file 1: Figure S1. Correlation between ND ratios of fathers and sons (n = 26 pairs). Figure S2. Selection population ND ratios after 5 months of selection relaxation. Distribution of ND ratios of males from the diurnal (D), nocturnal (N) and control (C) populations after 5 months (~ 15 generation) of selection relaxation. Average ND ± stdev values are reported for each line. The ND ratio of the N population was significantly different from both that of the control and D populations (Kolmogorov-Smirnov (KS) t-test for N vs C D = 0.56, p < 0.001, N vs D D = 0.68, p < 0.001). The ND ratios of the D and C populations were not significantly different (KS, D = 0.23, p = NS). Figure S3. Locomotor behaviour and sleep of the selection lines in LD conditions. A. LD acrophase angles of morning (MP) and evening (EP) peaks of activity for N (black circles, n = 230), D (red circles, n = 160) and C (blue triangles, n = 57) populations. Lines represent mean vectors±95%CI. One hour corresponds to a 15° angle. ZT0 and ZT12 are represented by 0° and 180° angles, respectively. The MP of N flies (n = 230) was significantly advanced, compared to that of both C (n = 57) and D (n = 160) flies, as tested by ANOVA (F2,444:163.87, p < 0.0001). The EP of N flies (n = 264) was significantly delayed, as compared to C (n = 82) and D (n = 170) flies; (F2,513:73.77, p < 0.0001). B. Total sleep, bins per hour shown for N (black, n = 304), D (red, n = 171) and C (blue, n = 100) populations. Data points correspond to averages ± SEM. The white/grey boxes represent day/night, respectively. N flies slept more during the day (n = 297) than did D (n = 166) and C (n = 96) flies (F11,2224 = 71.02, p < 0.0001). D flies slept more that did C and N flies during the night (F11,2232:93.45, p < 0.0001). There is no significant difference between N and C (TuskeyHSD, p = 0.08, NS) flies in terms of night sleep. C. The median locomotor activity per 30 min bin (±SEM) is shows for D (red), N (black) and C (b [file 12864_2020_7020_MOESM1_ESM.docx]

Supplemental Information

The genetic basis of diurnal preference in *Drosophila melanogaster*

Mirko Pegoraro, Laura M.M. Flavell, Pamela Menegazzi , Perrine Colmbi, Pauline Dao Charlotte Helfrich-Förster and Eran Tauber

Corresponding author: Eran Tauber

Email: [eran.tauber@gmail.con](mailto:eran.tauber@gmail.con)

**This PDF file includes:**

Figs. S1 to S8

Tables S1 to S5

Supplemental Methods

Supplemental References

**
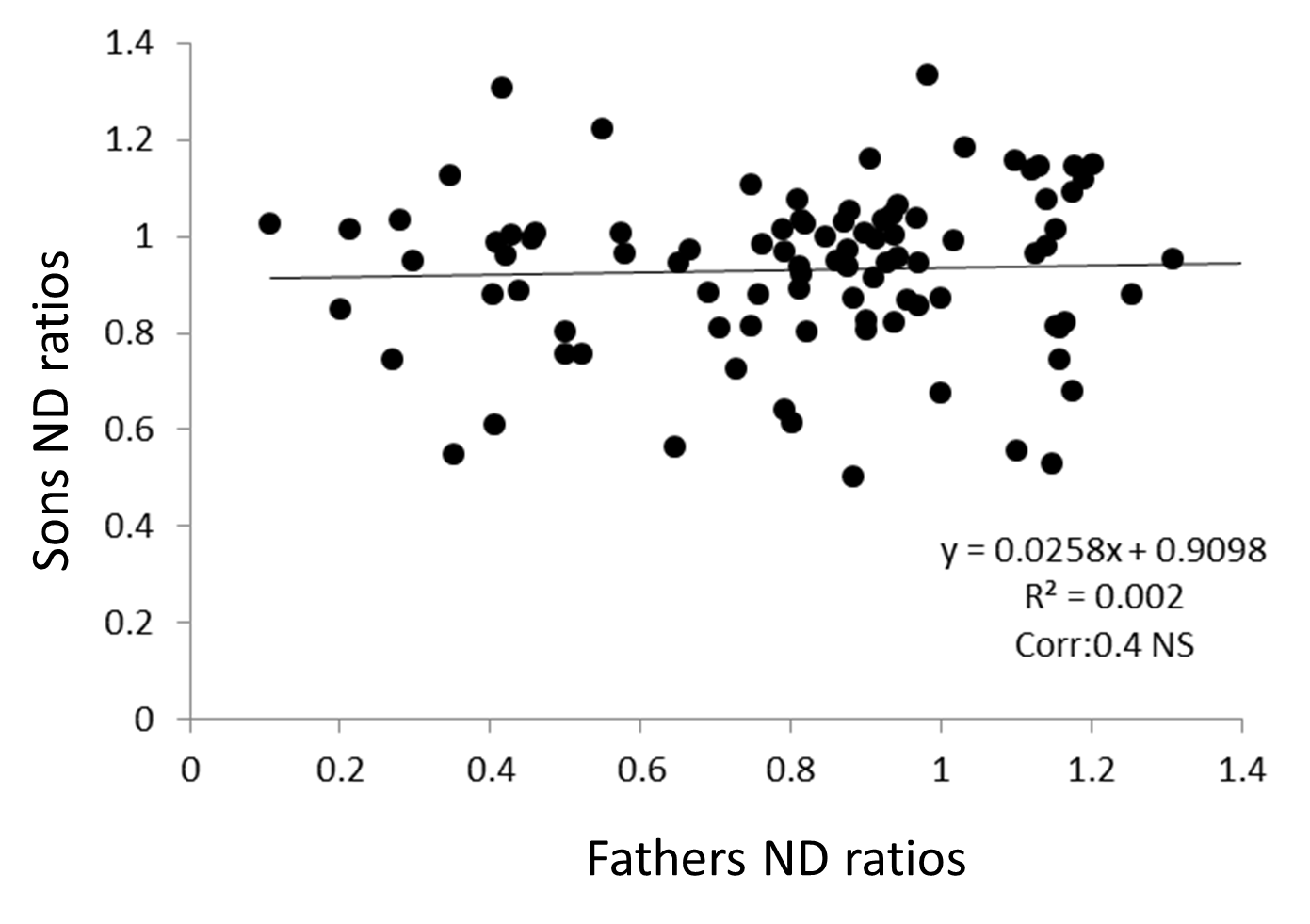
**

**Fig. S1**. **Correlation between ND ratios of fathers and sons (n = 26 pairs)**.


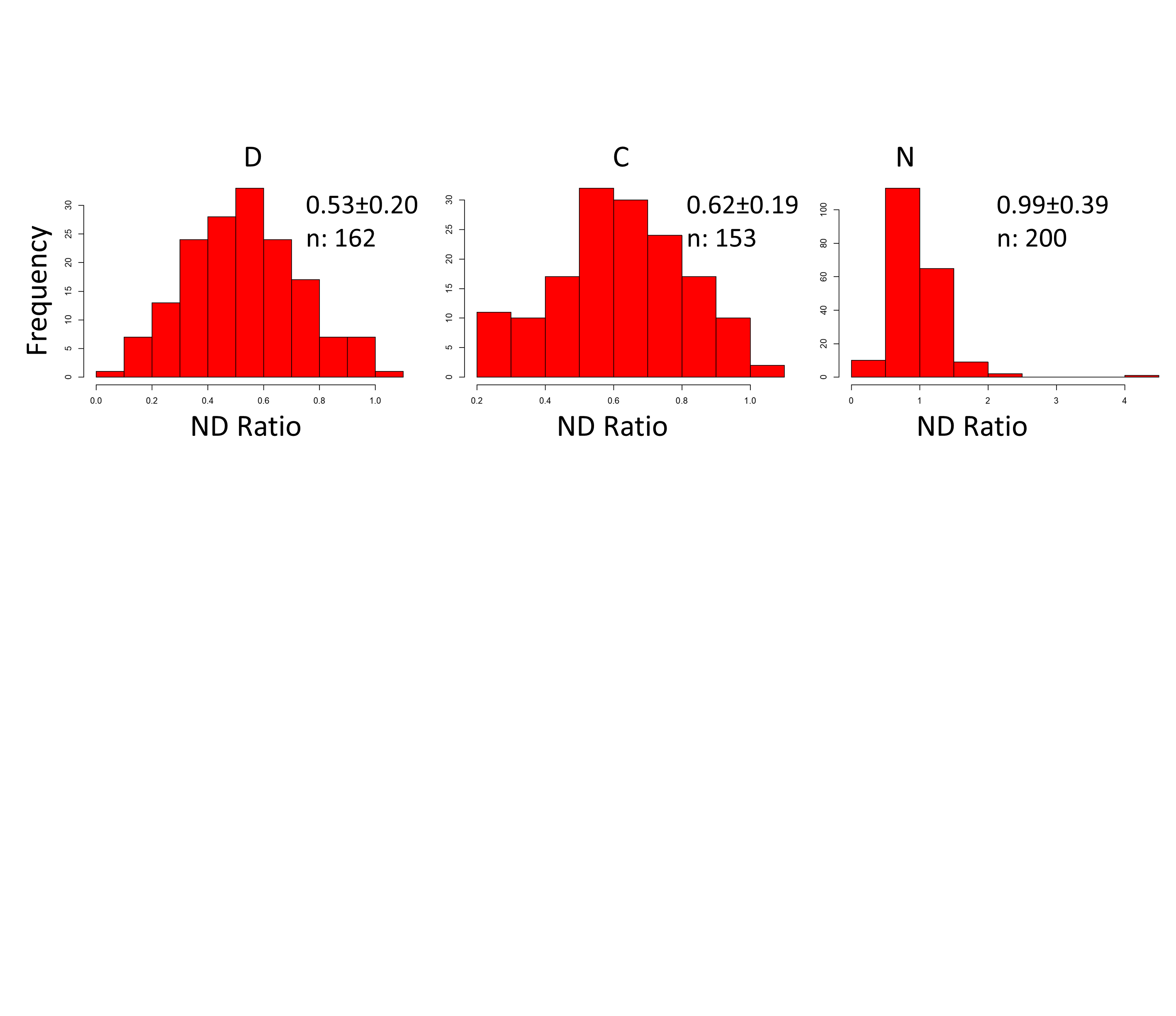


Fig. S2. **Selection population ND ratios after 5 months of selection relaxation**. Distribution of ND ratios for males from the diurnal (D), nocturnal (N) and control (C) populations after 5 months (~15 generation) of selection relaxation. Average ND ± stdev values are reported for each line. The ND ratio of the N population was significantly different from both that of the control and D populations (Kolmogorov-Smirnov (KS) t-test for N vs C D= 0.56, p<0.001, N vs D D= 0.68, p<0.001). The ND ratios of the D and C populations were not significantly different (KS, D= 0.23, p=NS).

**
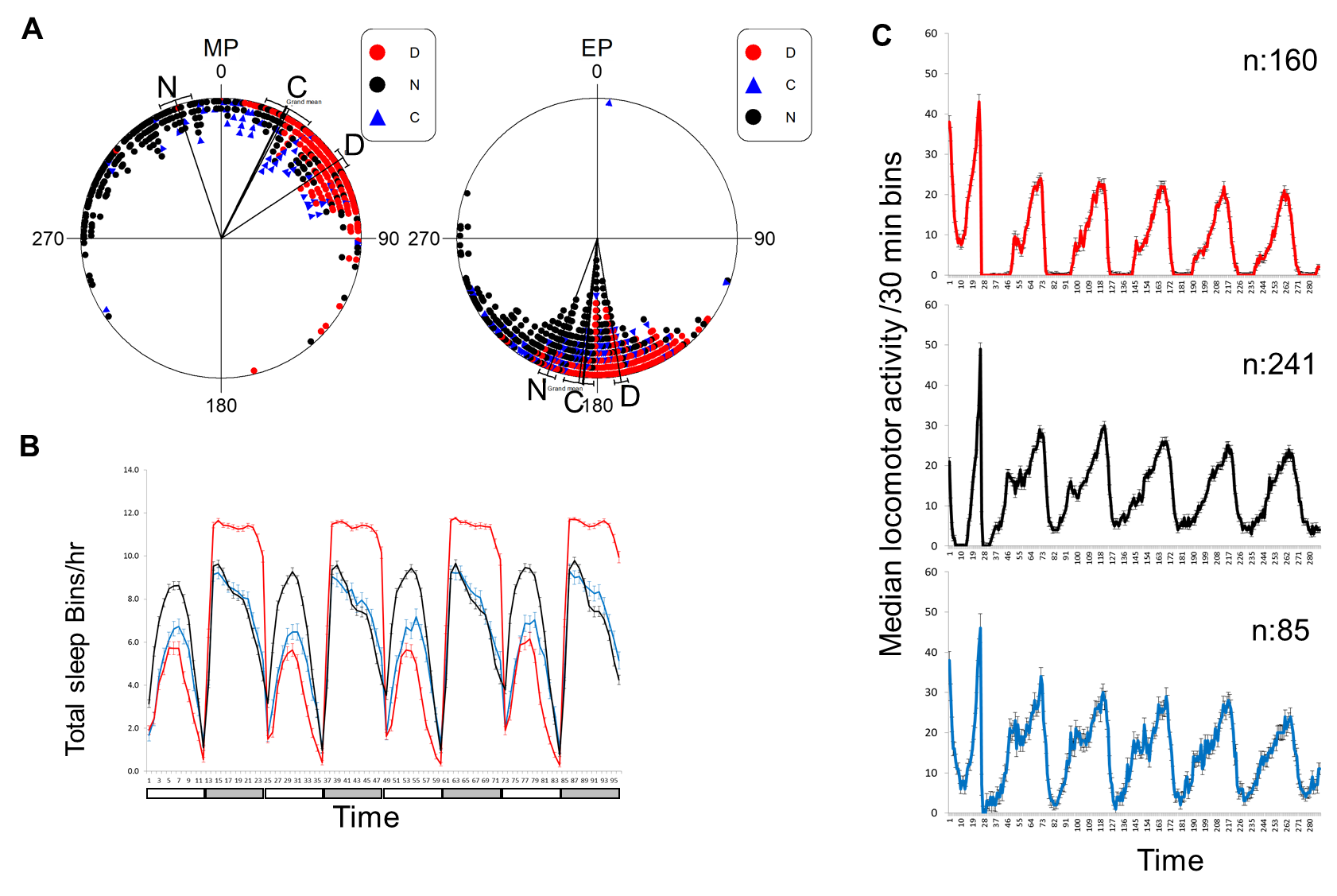
**

Fig. S3. **Locomotor behaviour and sleep of the selection lines in LD cycles**. **A.** LD acrophase angles of morning (MP) and evening (EP) peaks of activity for N (black circles, n=230), D (red circles, n=160) and C (blue triangles, n=57) populations. Lines represent mean vectors ± 95%CI. One hour corresponds to a 15° angle. ZT0 and ZT12 are represented by 0° and 180° angles, respectively. The MP of N flies (n=230) was significantly advanced, as compared to that of both C (n=57) and D (n=160) flies, as tested by ANOVA (F_2,444_:163.87, p<0.0001). The EP of N flies (n=264) was significantly delayed, as compared to those of C (n=82) and D (n=170) flies; (F_2,513_:73.77, p<0.0001). **B.** Total sleep, bins per hour shown for N (black, n=304), D (red, n=171) and C (blue, n =100) flies. Data points represent averages ± SEM. The white/grey boxes represent day/night, respectively. N flies sleep more during the day (n=297) than do D (n=166) and C (n=96) flies (F_11,2224_ = 71.02, p<0.0001). D flies sleep more that do C and N flies during the night (F_11,2232_:93.45, p<0.0001). There is no significant difference between N and C flies (TuskeyHSD, p=0.08, NS) in terms of night sleep. **C.** The median locomotor activity per 30 min bin (±SEM) is shown for D (red), N (black) and C (blue) flies during the last day in a LD 12:12 cycle and the first 5 days in constant conditions (DD cycle, 25°C). For each profile, the number of flies is depicted.

**
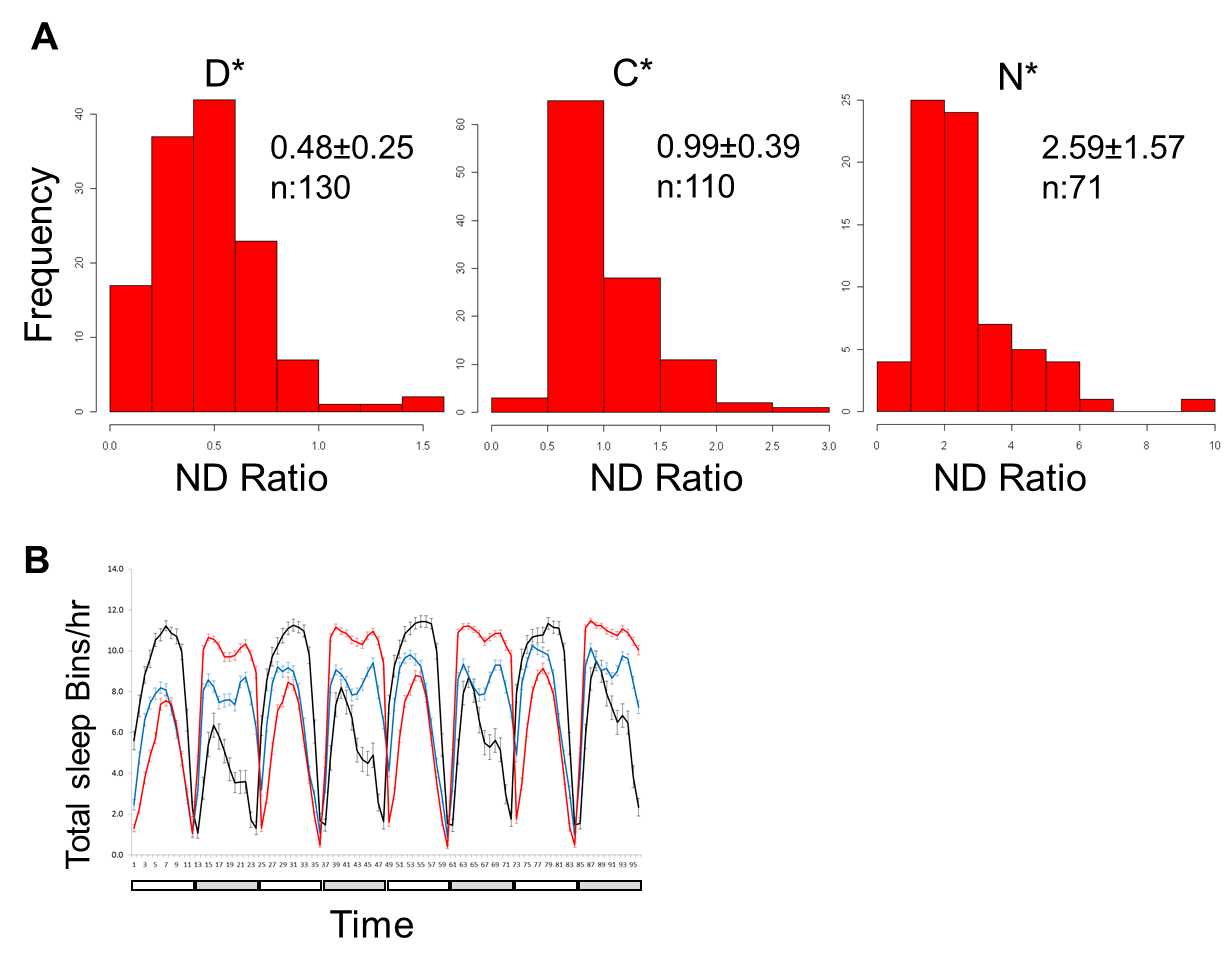
**

Fig. S4. **ND ratios and sleep of isogenic strains**. **A.** Distribution of ND ratios for males of the diurnal (D*), nocturnal (N*) and control (C*) isogenic lines. Average ND ± SD is reported for each line (note different X-axis scales). **B.** Total sleep bins per hour over time for the N* (black, n=70), D* (red, n=170) and C* (blue, n=130) isogenic lines. Data points are averages ± SEM. The white/grey boxes represent day/night, respectively.

**
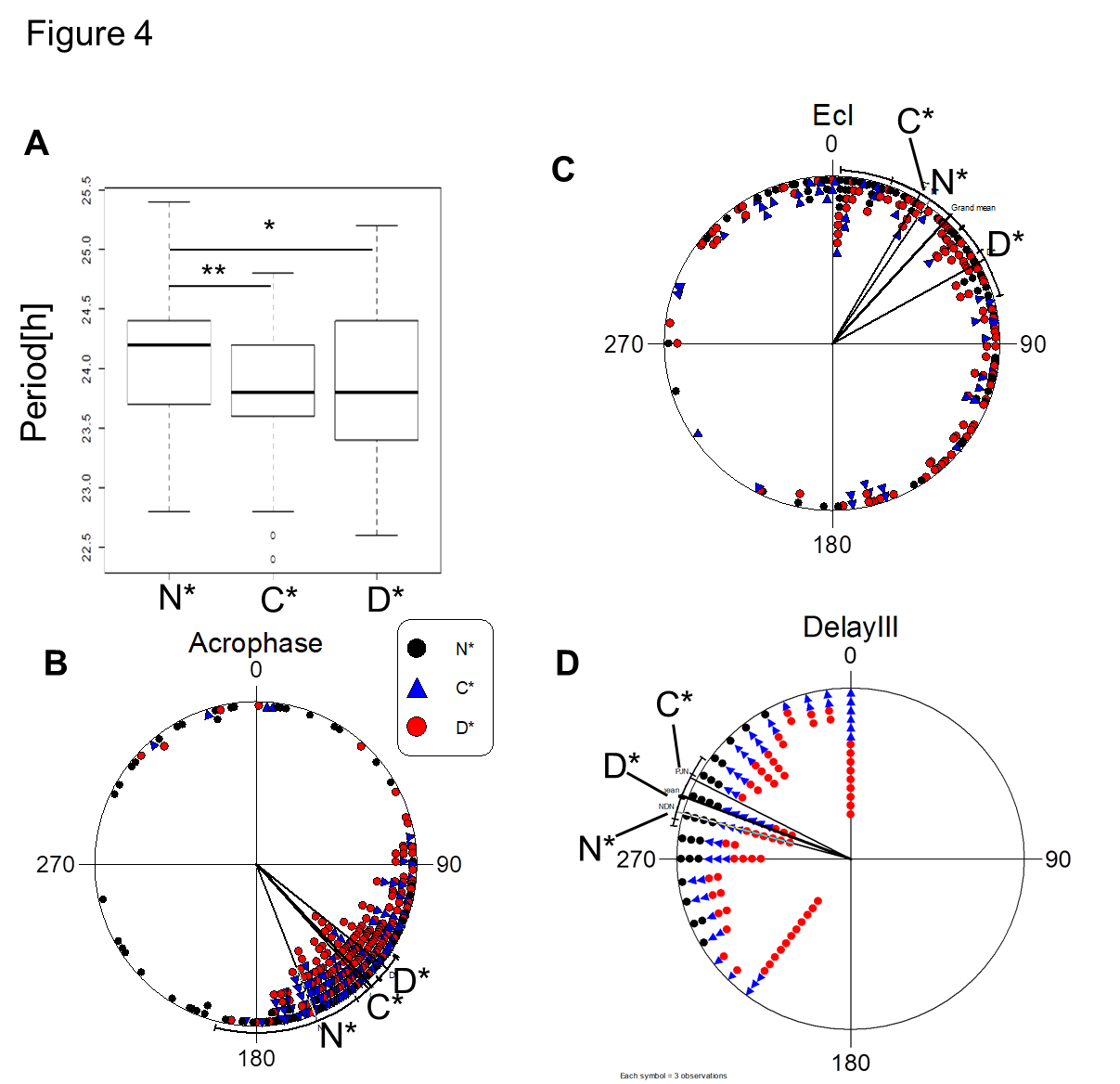
**

**Fig. S5. Circadian behaviour of isogenic strains (N*, D* and C*). A.** Boxplots of free-running periods of the N* (n=64), C* (n=124) and D* (n=157) isogenic lines. Solid lines represent median periods, the bottom and upper ends of the box correspond to the upper and lower quartiles, respectively, and the whiskers denote maximum and minimum values, excluding outliers. TukeyHSD test, *p<0.05, **p<0.001 **B.** Acrophase angles of the free-running activity shown for the N* (black circles, n=64), D* (red circles, n = 157) and C* (blue triangles, n=124) isogenic lines. The phase in the N* line was delayed by 2.02 h, as compared to what was measured in the D* line, and by 1.38 h, as compared to what was measured in the C* line (F_2,342_:6.01, p<0.01). Lines represent mean vectors ± 95% CI. One hour corresponds to a 15° angle. **C.** Phase of eclosion in the N* (black circle, n=75), D* (red circle, n=111) and C* (blue triangle, n=50) lines. The eclosion phase of D* flies was delayed by ~2 h, as compared to what was measured with both the N* and C* lines (F_2,233_:4.95, p<0.01). There was no difference between N* and C* flies (F_1,123_:0.08, p=0.78, NS). Lines represent mean vectors ± 95% CI. One hour corresponds to an angle of 15°. Light-on (ZT0) and light-off (ZT12) translated to 0° and 180° angles, respectively. **D.** Phase delays of N* (black circles, n = 66), D* (red circles, n=170) and C* (blue triangles, n=126) flies. There were no differences among the strains (F_2,359_:1.93, p = 0.15, NS). Lines represent mean vectors ± 95% CI.

**
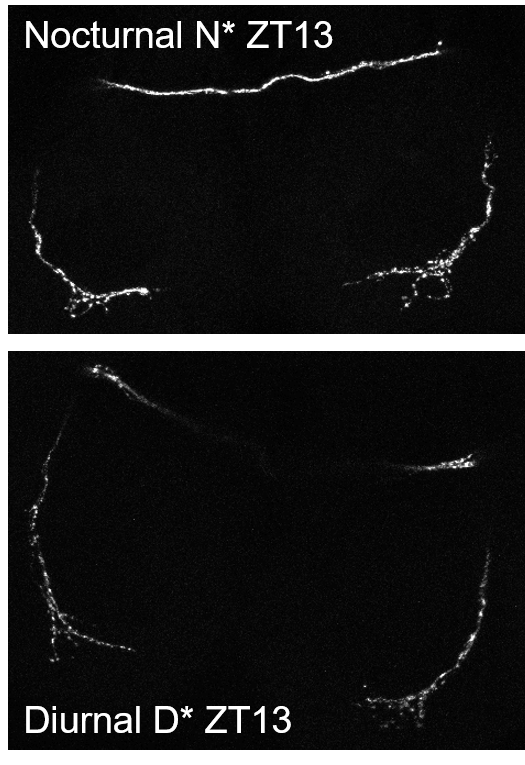
**

**Fig. S**6**.** **Representative PDF staining in LNv projections.** Representative PDF staining of LNv projections at ZT13 in N* (top) and D*(bottom) lines maintained in a LD12:12 cycle at 25°C.


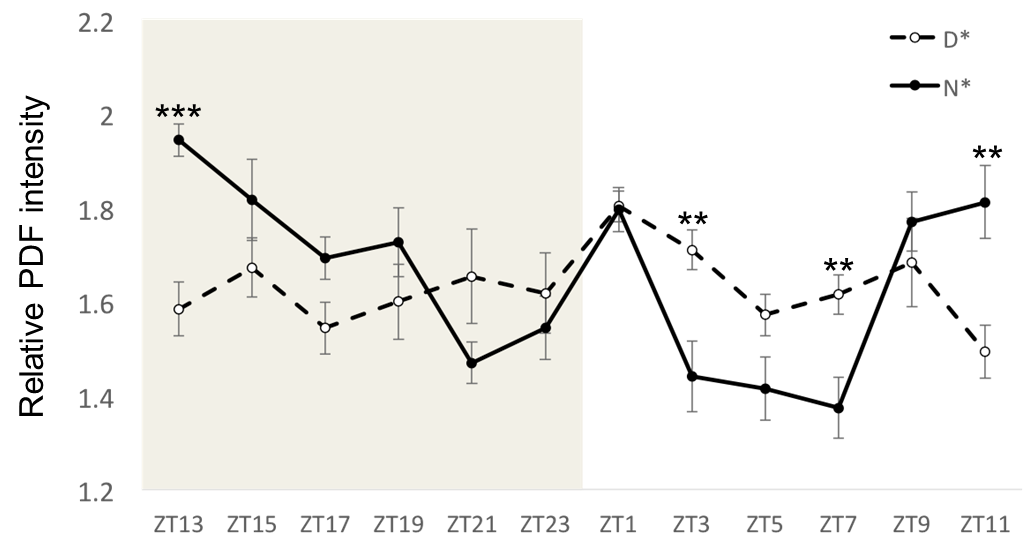


**Fig. S7.** **Expression of PDF in LNv projections.**  PDF staining in the N* (full lines) and D* (dashed lines) lines maintained in a 12:12 LD cycle. Shading represents light-off. Representative staining is shown in Supplementary Fig. S4. Points represent averages ± standard error. The N* signal was lower than the D* signal at ZT3 (F_1,18_=11.99, p<0.01) and ZT7 (F_1,19_=10.13, p<0.01), and higher at ZT11 (F_1,15_=10.53, p<0.01) and ZT13 (F_1,17_=23.39, p<0.001).


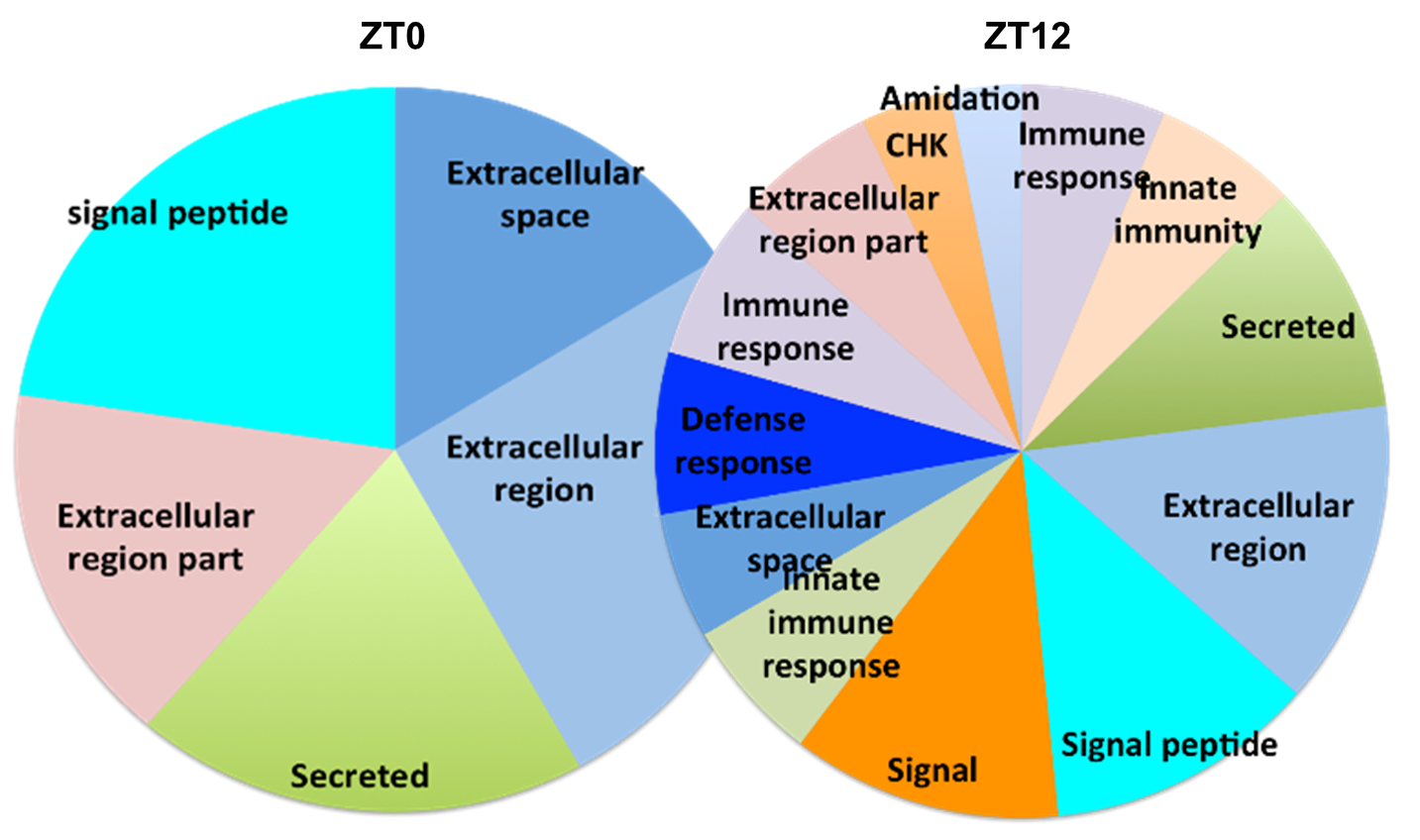


**Fig. S8.** **Functional annotation of DEGs associated with diurnal preference.** Pie charts representing significant terms of DEGs in 3 pairwise contrasts (D* vs N*, D* vs C*, N* vs C*) at ZT0 and ZT12. Sections represent the percent of enrichment for each term. p<0.05 after Benjamini correction with the exception of the “signal peptide” term at ZT0, where p = 0.054.

**Table S1. Artificial selection ND ratios and heritability**. ND ratios per cycle of selection (Cyc). Stdev indicates standard deviation and N is the number of rhythmic males. R is the response to selection and S is the selection differential per cycle of selection. Vp is variance of the ND ratio per cycle of selection and Var(h^2^) is the variance in h^2^ due to genetic drift. Cum R and Cum S correspond to the cumulative response to selection and the cumulative selection differential, respectively. KS D and pval are the results of the Kolmogorov-Smirnov test comparing ND ratios of two consecutive generations (n = 25 in all cases).

| **Selection** | **Cyc** | **average** | **stdev** | **N** | **R** | **S** | **h2** | **Vp** | **Var(h2)** | **Cum R** | **Cum S** | **KS D** | **KS pval** |
| --- | --- | --- | --- | --- | --- | --- | --- | --- | --- | --- | --- | --- | --- |
| Nocturnal | C0 | 0.88 | 0.21 | 176 |  |  |  |  |  | 0 | 0 |  |  |
|  | C1 | 1.04 | 0.29 | 183 | 0.15 | 0.52 | 0.29 | 0.09 | 0.007 | 0.15 | 0.52 | 0.35 | <0.0001 |
|  | C2 | 1.01 | 0.38 | 249 | -0.03 | 0.78 | 0.00 | 0.14 | 0.002 | 0.12 | 1.30 | 0.18 | <0.01 |
|  | C3 | 1.01 | 0.36 | 216 | 0.00 | 0.68 | 0.00 | 0.13 | 0.003 | 0.12 | 1.99 | 0.05 | 0.918 |
|  | C4 | 1.13 | 0.34 | 223 | 0.12 | 0.67 | 0.19 | 0.12 | 0.004 | 0.25 | 2.66 | 0.20 | <0.0001 |
|  | C5 | 1.10 | 0.24 | 160 | -0.03 | 0.36 | 0.00 | 0.06 | 0.005 | 0.22 | 3.02 | 0.10 | 0.294 |
|  | C6 | 1.23 | 0.40 | 208 | 0.12 | 0.76 | 0.16 | 0.16 | 0.004 | 0.34 | 3.79 | 0.20 | <0.01 |
|  | C7 | 1.24 | 0.37 | 238 | 0.02 | 0.76 | 0.02 | 0.14 | 0.002 | 0.36 | 4.55 | 0.06 | 0.773 |
|  | C8 | 1.17 | 0.39 | 147 | -0.07 | 0.47 | 0.00 | 0.15 | 0.009 | 0.29 | 5.03 | 0.15 | <0.05 |
|  | C9 | 1.09 | 0.46 | 220 | -0.09 | 0.46 | 0.00 | 0.21 | 0.009 | 0.20 | 5.48 | 0.16 | <0.05 |
|  | C10 | 1.20 | 0.41 | 215 | 0.11 | 0.84 | 0.13 | 0.17 | 0.003 | 0.31 | 6.32 | 0.19 | <0.01 |
|  |  |  |  |  |  |  |  |  |  |  |  |  |  |
| **Selection** | **Cyc** | **average** | **stdev** | **N** | **R** | **S** | **h2** | **Vp** | **Var(h2**) | **Cum R** | **Cum** **S** | **KS D** | **KS pval** |
| Diurnal | C0 | 0.88 | 0.21 | 176 |  |  |  |  |  | 0.00 | 0.00 |  |  |
|  | C1 | 0.81 | 0.21 | 104 | -0.07 | -0.30 | 0.25 | 0.04 | 0.015 | -0.07 | -0.30 | 0.22 | <0.01 |
|  | C2 | 0.84 | 0.23 | 226 | 0.03 | -0.36 | 0.00 | 0.05 | 0.004 | -0.04 | -0.65 | 0.06 | 0.911 |
|  | C3 | 0.76 | 0.21 | 164 | -0.08 | -0.35 | 0.21 | 0.04 | 0.008 | -0.12 | -1.01 | 0.12 | 0.098 |
|  | C4 | 0.75 | 0.22 | 284 | -0.02 | -0.44 | 0.04 | 0.05 | 0.002 | -0.14 | -1.44 | 0.06 | 0.858 |
|  | C5 | 0.68 | 0.24 | 233 | -0.07 | -0.42 | 0.16 | 0.06 | 0.005 | -0.20 | -1.86 | 0.14 | <0.01 |
|  | C6 | 0.41 | 0.18 | 263 | -0.27 | -0.26 | 1.03 | 0.03 | 0.024 | -0.47 | -2.12 | 0.49 | <0.0001 |
|  | C7 | 0.53 | 0.24 | 223 | 0.12 | -0.33 | 0.00 | 0.06 | 0.005 | -0.35 | -2.45 | 0.28 | <0.0001 |
|  | C8 | 0.44 | 0.22 | 274 | -0.10 | -0.20 | 0.48 | 0.03 | 0.019 | -0.45 | -2.66 | 0.23 | <0.0001 |
|  | C9 | 0.33 | 0.20 | 316 | -0.10 | -0.24 | 0.44 | 0.04 | 0.017 | -0.55 | -2.89 | 0.25 | <0.0001 |
|  | C10 | 0.32 | 0.20 | 221 | -0.01 | -0.21 | 0.05 | 0.04 | 0.010 | -0.56 | -3.11 | 0.07 | 0.568 |
|  |  |  |  |  |  |  |  |  |  |  |  |  |  |
| **Selection** | **Cyc** | **average** | **Stdev** | **N** |  |  |  |  |  |  |  |  |  |
| Control | C0 | 0.88 | 0.21 | 176 |  |  |  |  |  |  |  |  |  |
|  | C4 | 0.85 | 0.15 | 19 |  |  |  |  |  |  |  |  |  |
|  | C10 | 0.77 | 0.25 | 78 |  |  |  |  |  |  |  |  |  |
|  |  |  |  |  |  |  |  |  |  |  |  |  |  |

**Table S2.** List of isofemale strains used to generate the synthetic population.

| **Name** | **n** | **Collection region** | **Latitude/**  **Longitude** | **Collection**  **year** |
| --- | --- | --- | --- | --- |
| MAR | 57 | Market Harborough (UK) | 52.478/-0.921 | 2008 |
| HOJ | 20 | Hojbjeng (Denmark) | 56.114/10.190 | 2009 |
| DJ | 1 | Dijon (France) | 47.321/5.041 | 2000 |
| KOR | 13 | Korpilahti (Finland) | 62.043/25.580 | 2008 |
| BIT | 7 | Bitetto (Italy) | 41.041/16.749 | 2004 |
| M-REN | 18 | Rende (Italy) | 39.332/16.184 | 2006 |
| COR | 4 | Corces (Italy) | 46.633/10.763 | 2004 |
| RUT | 8 | Rutigliano (Italy) | 41.011/17.005 | 2004 |
| CORSTA | 2 | Corces st. Agidius (Italy) | 46.633/10.763 | 2004 |
| NAT | 3 | Naturno (Italy) | 46.650/11.004 | 2004 |
| FUL | 2 | Fulda (Germany) | 50.552/9.676 |  |
| POS | 3 | Postal (Italy) | 46.607/11.193 | 2004 |
| KIL | 77 | Kilworth (UK) | 52.446/-1.078 | 2008 |
| STO | 8 | Stockholm (Sweden) | 59.325/18.071 | 2008 |
| KNO | 6 | Knossos (Creete) | 35.298/25.163 | 2002 |
| BUR | 9 | Burgundy (France) | 47.278/4.222 | 2000 |
| HU | 4 | Huten (Holland) |  | 2000 |
| GOT | 7 | Goteborg (Sweden) | 57.707/11.967 | 2008 |
| SAL | 1 | Salice (Italy) | 39.706/15.812 | 2004 |
| NOV | 1 | Noventa (Italy) | 45.291/11.540 | 2004 |
| VIL | 3 | Villorba (Italy) | 45.729/12.256 | 2004 |
| LEO | 1 | St. Leonhard (Italy) | 46.719/11.696 | 2004 |
| NN | 2 |  |  |  |
| ZS | 1 | Sengwa (Zimbabwe) | -17.671/28.240 | from David Dolezel (originally from Shu Fang) |
| ZH | 1 | Harare (Zimbabwe) | -17.832/31.046 | from David Dolezel (originally from Shu Fang) |
| CAV | 4 | Cavarzere (Italy) | 45.136/12.081 | 2004 |
| AFRW | 1 | Africa |  |  |
| B16 | 1 |  |  |  |
| NAT | 1 | Naturno (Italy) | 46.650/11.004 | 2004 |
| LOR | 1 | St. Lorenz (Italy) | 46.460/11.804 |  |
| BAD | 1 | Bad Salzschlirf (Germany) | 50.624/9.507 | 2004 |
| LE | 3 | Leiden (Nederland) | 52.152/4.481 | 2000 |
| CORF | 1 | Corbires-Fribourg (Switzerland) | 46.658/7.108 | 2004 |

**Table S3** List of differentially expressed genes (p<0.05) in all pairwise contrasts. A group of 34 genes were differentially expressed both at ZT0 and ZT12 (**ZT0 ZT12**). Nineteen genes were uniquely identified at ZT0 (**ZT0)** and 87 were differentially expressed only at ZT12 (**ZT12**). Unknown transcripts are indicated by the suffix TCONS.

| **ZT0 ZT12** | **ZT0** | **ZT12** | | |
| --- | --- | --- | --- | --- |
| Lsp1beta | CG10680 | nAcRbeta-21C | CG3590 | CecA1 |
| Tps1 | CG9928 | Cyp28d1 | Pdf | CecA2 |
| nimB2 | CG15293 | Cpr | CG9691 | CG18673 |
| IM4 | CG6543 | CG9505 | regucalcin | Ugt35b |
| Irc | Amy-p | CG8665 | CG31743 | CG18765 |
| CG14273 | CG15068 | CG9498 | Cpr47Ee | CG10513 |
| CG6012 | Mst98Cb | CG16978 | Ipk1 | CG1681 |
| Cyp6w1 | CG9759 | Amy-d | CG16898 | CG15347 |
| CG34227 | CG10553 | IM2 | CG6188 | TCONS_00015278 |
| CG11395 | msta | IM14 | CG11317 | TCONS_00020522 |
| CG16836 | CG9377 | Cyp9c1 | Cpr11B | TCONS_00029494 |
| Cyp9b2 | Dro | CG8216 | Sr-CI |  |
| CG13335 | Nplp2 | Ance-4 | CG34172 |  |
| CG30083 | CG7402 | CG15117 | CG15414 |  |
| CG17574 | CG7678 | GV1 | CG12512 |  |
| CG8329 | Obp99b | CG42324 | CG7532 |  |
| CG7470 | CG2233 | CG32368 | CG30002 |  |
| dAtg1 | TCONS_00013524 | LanB2 | CG30203 |  |
| Cpr72Ec | TCONS_00013630 | CG9701 | Uhg5 |  |
| Ccp84Ag |  | Lcp65Ag2 | Uhg1 |  |
| CG31157 |  | pst | GstE2 |  |
| TotC |  | Ilp3 | GstE8 |  |
| CG31326 |  | Pdh | sano |  |
| CG6687 |  | CG4306 | NtR |  |
| Rh3 |  | CG12947 | CG4269 |  |
| CG10550 |  | CG4757 | CG4250 |  |
| CG11909 |  | TotA | CG7722 |  |
| toy |  | CG5791 | Cyp12d1-p |  |
| CG4064 |  | Lsd-1 | CG16926 |  |
| CG9360 |  | Ela | CG12362 |  |
| Act79B |  | to | Pdp1 |  |
| TCONS_00015283 |  | CG5527 | CG14105 |  |
| TCONS_00027521 |  | CG11841 | Adgf-A |  |
| TCONS_00035728 |  | CG11842 | GNBP2 |  |
|  |  | CG5195 | CG4525 |  |
|  |  | CG7202 | Rh2 |  |
|  |  | CG8319 | CG5849 |  |
|  |  | CG14257 | CG7054 |  |

**Table S4** Differentially expressed genes (p<0.05). Biological processes are as reported in flybase (http://flybase.org/).

| **Direction of expression** | **Symbol** | **Annotation symbol** | **Time Points** | **Biological process** |
| --- | --- | --- | --- | --- |
| D*>C*>N* | Tps1 | CG4104 | ZT0, ZT12 | Photoreceptor cell maintenance; trehalose biosynthetic process. |
|  | nimB2 | CG31839 | ZT0 | Mesoderm development; defense response to bacterium. |
|  | Cyp9b2 | CG4486 | ZT0, ZT12 | Oxidation-reduction process. |
|  | Cyp9c1 | CG3616 | ZT12 | Oxidation-reduction process. |
|  | Amy-p | CG18730 | ZT0 | Sleep; carbohydrate metabolic process. |
|  | Irc | CG8913 | ZT0, ZT12 | Response to oxidative stress |
|  | TotA | CG31509 | ZT12 | Cellular response to UV; cellular response to oxidative stress. |
|  | TotC | CG31508 | ZT0, ZT12 | Cellular response to heat; cellular response to UV; |
|  | Rh3 | CG10888 | ZT0, ZT12 | Phototransduction, UV; visual perception. |
|  | nAcRbeta-21C | CG11822 | ZT12 | Synaptic transmission, cholinergic; sleep. |
|  | Cpr | CG11567 | ZT12 | Oxidation-reduction process. |
|  | Ance-4 | CG8196 | ZT12 | Proteolysis. |
|  | pst | CG8588 | ZT12 | Protein secretion; learning or memory. |
|  | Ilp3 | CG14167 | ZT12 | Female mating behavior; sleep; insulin receptor signaling pathway; locomotor rhythm. |
|  | Ela |  | ZT12 | Unknown |
|  | Uhg1 | CR32886 | ZT12 | Unknown |
|  | Pdp1 | CG17888 | ZT12 | Mitotic nuclear division; positive regulation of transcription; circadian rhythm; regulation of lipid metabolic process; DNA endoreduplication; growth; response to nutrient. |
|  | Adgf-A | CG5992 | ZT12 | Cell proliferation; adenosine catabolic process; cellular response to sucrose starvation; larval lymph gland hemopoiesis; hemocyte differentiation; regulation of carbohydrate metabolic process. |
|  | Gbp2 | CG11395 | ZT0, ZT12 | Signal transduction |
|  | Vha100-4 | CG7678 | ZT0 | ATP hydrolysis coupled proton transport. |
|  | AdSL | CG3590 | ZT12 | Purine ribonucleotide biosynthetic process; purine nucleotide metabolic process. |
|  | atk | CG5195 | ZT12 | Locomotion; cilium morphogenesis; sensory organ development. |
|  | CG9505 | CG9505 | ZT12 | Proteolysis. |
|  | CG9498 | CG9498 | ZT12 | Unknown |
|  | CG16978 | CG16978 | ZT12 | Unknown |
|  | CG14273 | CG14273 | ZT0, ZT12 | Unknown |
|  | CG34227 | CG34227 | ZT0, ZT12 | Unknown |
|  | CG13335 | CG13335 | ZT0 | Unknown |
|  | CG17574 | CG17574 | ZT0, ZT12 | Unknown |
|  | CG7470 | CG7470 | ZT0, ZT12 | Epithelium development; germarium-derived egg chamber formation; proline biosynthetic process; oxidation-reduction process. |
|  | CG9759 | CG9759 | ZT0 | Unknown |
|  | CG31326 | CG31326 | ZT0, ZT12 | Unknown |
|  | CG15117 | CG15117 | ZT12 | Multicellular organism reproduction; carbohydrate metabolic process. |
|  | CG9701 | CG9701 | ZT12 | Carbohydrate metabolic process. |
|  | CG5527 | CG5527 | ZT12 | Proteolysis. |
|  | CG11317 | CG11317 | ZT12 | Neurogenesis. |
|  | CG7202 | CG7202 | ZT12 | Unknown |
|  | CG5849 | CG5849 | ZT12 | Proteolysis. |
|  |  |  |  |  |
| **Direction of expression** | **Symbol** | **Annotation symbol** | **Time Points** | **Biological process** |
| N*>C*>D* | Dro | CG10816 | ZT0 | Defense response to bacterium; defense response to Gram-positive bacterium. |
|  | dATG1 | CG10967 | ZT0, ZT12 | Response to oxidative stress; negative regulation of signaling; instar larval or pupal morphogenesis; organic substance catabolic process;; negative regulation of intracellular signal transduction; regulation of polysaccharide metabolic process. |
|  | Ccp84Ag | CG2342 | ZT0, ZT12 | Chitin-based cuticle development. |
|  | Cpr47Ee | CG13222 | ZT12 | Chitin-based cuticle development. |
|  | GV1 | CG12023 | ZT12 | Unknown |
|  | Lcp65Ag2 | CG10534 | ZT12 | Chitin-based cuticle development. |
|  | Pdh | CG4899 | ZT12 | Retinal metabolic process; phagocytosis; oxidation-reduction process. |
|  | to | CG11853 | ZT12 | Circadian rhythm; adult feeding behavior; mating behavior; male courtship behavior. |
|  | Pdf | CG6496 | ZT12 | Response to abiotic stimulus; single-organism behavior; positive regulation of circadian rhythm; reproduction; taxis; sensory perception; regulation of metabolic process; system process; positive regulation of circadian sleep/wake cycle, wakefulness. |
|  | GstE2 | CG17523 | ZT12 | Glutathione metabolic process. |
|  | GstE8 | CG17533 | ZT12 | Glutathione metabolic process. |
|  | Act79B | CG7478 | ZT12 | Cytoskeleton organization. |
|  | Ugt35b | CG6649 | ZT12 | UDP-glucose metabolic process. |
|  | Rh2 | CG16740 | ZT12 | Phototransduction; G-protein coupled receptor signaling pathway; visual perception. |
|  | GNBP2 | CG4144 | ZT12 | Regulation of innate immune response; carbohydrate metabolic process. |
|  | l(2)34Fc | CG7532 | ZT12 | Unknown |
|  | GstT4 | CG1681 | ZT12 | Unknown |
|  | CG8329 | CG8329 | ZT0, ZT12 | Sleep; proteolysis. |
|  | CG31157 | CG31157 | ZT0, ZT12 | Unknown |
|  | CG9360 | CG9360 | ZT0, ZT12 | Unknown |
|  | CG8665 | CG8665 | ZT12 | 10-formyltetrahydrofolate catabolic process; one-carbon metabolic process; oxidation-reduction process; biosynthetic process. |
|  | CG42324 | CG42324 | ZT12 | Unknown |
|  | CG12947 | CG12947 | ZT12 | Unknown |
|  | CG9691 | CG9691 | ZT12 | Unknown |
|  | CG34172 | CG34172 | ZT12 | Unknown |
|  | CG12512 | CG12512 | ZT12 | Metabolic process. |
|  | CG12362 | CG12362 | ZT12 | Protein ubiquitination involved in ubiquitin-dependent protein catabolic process. |
|  | CG4525 | CG4525 | ZT12 | Cilium assembly. |
|  | CG7054 | CG7054 | ZT12 | Unknown |
|  | CG14257 | CG14257 | ZT12 | Unknown |
|  | CG18765 | CG18765 | ZT12 | Unknown |

**Table S5** Complementation tests. Average ND ratios and standard deviation for each complementation test cross are shown. “n” indicates the number of flies tested. Kolmogorov-Smirnov tests (KS-test) are reported as matrixes with p values in the top half and D values in bottom half [1,2]. Kruskal-Wallis rank sum test (KW-test) results with 2 degree of freedom are also shown for each complementation test [1,2]. Compl indicates the result of the complementation test. Positive complementation was confirmed only for the ND D*cross<C*cross<N*cross. The KS-test was significant for the N*cross vs D*cross contrast and the KW-test was also significant.

|  |  |  |  | **Ks-test** | | | **Kruskal-Wallis** | | **Compl** |
| --- | --- | --- | --- | --- | --- | --- | --- | --- | --- |
| **gen** | **average** | **stdev** | **n** | **D*** | **C*** | **N*** | **Chi-sq** | **p-value** |  |
| *Ilp6^41^/D** | 0.475 | 0.279 | 30 | \|\|\|\|\|\|\| | <0.05 | <0.0001 | 21.375 | <0.0001 | Positive |
| *Ilp6^41^/C** | 0.566 | 0.220 | 32 | 0.3958 | \|\|\|\|\|\|\| | 0.073 |  |  |  |
| *Ilp6^41^/N** | 0.685 | 0.178 | 31 | 0.6409 | 0.3125 | \|\|\|\|\|\|\| |  |  |  |
| *para^MB08120^/D** | 0.596 | 0.197 | 32 | \|\|\|\|\|\|\| | 0.069 | <0.0001 | 49.028 | <0.0001 | Positive |
| *para^MB08120^/C** | 0.701 | 0.160 | 32 | 0.3125 | \|\|\|\|\|\|\| | <0.0001 |  |  |  |
| *para^MB08120^/N** | 1.164 | 0.294 | 31 | 0.8075 | 0.745 | \|\|\|\|\|\|\| |  |  |  |
| *para^KG01807^/D** | 0.432 | 0.161 | 63 | \|\|\|\|\|\|\| | <0.05 | <0.0001 | 68.396 | <0.0001 | Positive |
| *para^KG01807^/C** | 0.518 | 0.182 | 64 | 0.2631 | \|\|\|\|\|\|\| | <0.0001 |  |  |  |
| *para^KG01807^/N** | 0.745 | 0.225 | 80 | 0.6187 | 0.4594 | \|\|\|\|\|\|\| |  |  |  |
| *Pdfr^5304^/D** | 0.510 | 0.199 | 31 | \|\|\|\|\|\|\| | 0.612 | <0.0001 | 26.339 | <0.0001 | Positive |
| *Pdfr^5304^/C** | 0.564 | 0.143 | 31 | 0.1845 | \|\|\|\|\|\|\| | <0.0001 |  |  |  |
| *Pdfr^5304^/N** | 0.804 | 0.217 | 32 | 0.5938 | 0.5313 | \|\|\|\|\|\|\| |  |  |  |
| *Pdfr^3369^/D** | 0.459 | 0.190 | 28 | \|\|\|\|\|\|\| | 0.72 | <0.01 | 12.890 | <0.01 | Positive |
| *Pdfr^3369^/C** | 0.448 | 0.129 | 28 | 0.1786 | \|\|\|\|\|\|\| | <0.01 |  |  |  |
| *Pdfr^3369^/N** | 0.591 | 0.163 | 30 | 0.4357 | 0.5143 | \|\|\|\|\|\|\| |  |  |  |
| *cry^02^/D** | 0.315 | 0.153 | 42 | \|\|\|\|\|\|\| | <0.0001 | <0.0001 | 86.693 | <0.0001 | Positive |
| *cry^02^/C** | 0.893 | 0.235 | 57 | 0.8534 | \|\|\|\|\|\|\| | <0.05 |  |  |  |
| *cry^02^/N** | 0.990 | 0.261 | 59 | 0.8644 | 0.2774 | \|\|\|\|\|\|\| |  |  |  |
| *per^01^/D** | 0.574 | 0.158 | 31 | \|\|\|\|\|\|\| | <0.05 | <0.0001 | 32.218 | <0.0001 | Positive |
| *per^01^/C** | 0.704 | 0.201 | 50 | 0.3342 | \|\|\|\|\|\|\| | <0.0001 |  |  |  |
| *per^01^/N** | 0.933 | 0.419 | 58 | 0.6207 | 0.4234 | \|\|\|\|\|\|\| |  |  |  |
| *na ^1^/D** | 0.356 | 0.356 | 23 | \|\|\|\|\|\|\| | <0.0001 | <0.0001 | 36.379 | <0.0001 | Positive |
| *na^1^/C** | 0.626 | 0.269 | 61 | 0.5046 | \|\|\|\|\|\|\| | <0.0001 |  |  |  |
| *na^1^/N** | 0.969 | 0.969 | 14 | 0.8696 | 0.5995 | \|\|\|\|\|\|\| |  |  |  |
| *msn^102^/D** | 0.382 | 0.173 | 32 | \|\|\|\|\|\|\| | <0.01 | <0.0001 | 47.297 | <0.0001 | Positive |
| *msn^102^/C** | 0.641 | 0.207 | 31 | 0.6313 | \|\|\|\|\|\|\| | <0.0001 |  |  |  |
| *msn^102^/N** | 1.083 | 0.817 | 32 | 0.8125 | 0.4304 | \|\|\|\|\|\|\| |  |  |  |
| *cora^5^/D** | 0.356 | 0.225 | 30 | \|\|\|\|\|\|\| | 0.136 | <0.01 | 15.032 | <0.001 | Positive |
| *cora^5^/C** | 0.479 | 0.308 | 29 | 0.2908 | \|\|\|\|\|\|\| | <0.05 |  |  |  |
| *cora^5^/N** | 0.631 | 0.287 | 30 | 0.5 | 0.3506 | \|\|\|\|\|\|\| |  |  |  |
| *Pdf^01^/D** | 0.671 | 0.193 | 26 | \|\|\|\|\|\|\| | <0.0001 | <0.0001 | 58.667 | <0.0001 | Positive |
| *Pdf^01^/C** | 0.464 | 0.227 | 48 | 0.4856 | \|\|\|\|\|\|\| | <0.0001 |  |  |  |
| *Pdf^01^/N** | 0.892 | 0.247 | 59 | 0.4654 | 0.6522 | \|\|\|\|\|\|\| |  |  |  |
| *Arr2^3^/D** | 0.563 | 0.198 | 30 | \|\|\|\|\|\|\| | <0.05 | 0.317 | 8.410 | <0.05 | Negative |
| *Arr2^3^/C** | 0.870 | 0.366 | 10 | 0.5333 | \|\|\|\|\|\|\| | <0.05 |  |  |  |
| *Arr2^3^/N** | 0.647 | 0.196 | 31 | 0.2366 | 0.4742 | \|\|\|\|\|\|\| |  |  |  |
| *chico^KG00032^/D** | 0.355 | 0.107 | 29 | \|\|\|\|\|\|\| | <0.01 | <0.01 | 20.333 | <0.0001 | Negative |
| *chico^KG00032^/C** | 0.589 | 0.220 | 25 | 0.5131 | \|\|\|\|\|\|\| | 0.095 |  |  |  |
| *chico^KG00032^/N** | 0.485 | 0.156 | 31 | 0.4549 | 0.3187 | \|\|\|\|\|\|\| |  |  |  |
| *Clk^ar^/D** | 0.883 | 0.173 | 31 | \|\|\|\|\|\|\| | <0.01 | <0.05 | 9.606 | <0.01 | Negative |
| *ClK^ar^/C** | 0.754 | 0.247 | 31 | 0.4839 | \|\|\|\|\|\|\| | 0.216 |  |  |  |
| *Clk^ar^/N** | 0.830 | 0.120 | 31 | 0.3548 | 0.2581 | \|\|\|\|\|\|\| |  |  |  |
| *Clk^out^/D** | 0.587 | 0.196 | 30 | \|\|\|\|\|\|\| | <0.0001 | <0.05 | 18.600 | <0.0001 | Negative |
| *Clk^out^/C** | 0.943 | 0.346 | 21 | 0.6476 | \|\|\|\|\|\|\| | <0.05 |  |  |  |
| *Clk^out^ /N** | 0.743 | 0.219 | 32 | 0.3625 | 0.3988 | \|\|\|\|\|\|\| |  |  |  |
| *Clk^jrk^/D** | 1.117 | 0.241 | 30 | \|\|\|\|\|\|\| | <0.01 | 0.356 | 5.869 | 0.053 | Negative |
| *Clk^jrk^/C** | 0.996 | 0.488 | 31 | 0.4484 | \|\|\|\|\|\|\| | <0.0001 |  |  |  |
| *Clk^jrk^/N** | 1.094 | 0.206 | 32 | 0.2271 | 0.5504 | \|\|\|\|\|\|\| |  |  |  |
| *fok^EY11126^/D** | 0.422 | 0.273 | 20 | \|\|\|\|\|\|\| | 0.546 | 0.288 | 1.333 | 0.514 | Negative |
| *fok^EY11126^/C** | 0.434 | 0.185 | 13 | 0.2692 | \|\|\|\|\|\|\| | 0.461 |  |  |  |
| *fok^EY11126^/N** | 0.478 | 0.237 | 32 | 0.2688 | 0.2668 | \|\|\|\|\|\|\| |  |  |  |
| *fok^KG05913^/D** | 0.388 | 0.135 | 31 | \|\|\|\|\|\|\| | 0.559 | <0.05 | 2.503 | 0.286 | Negative |
| *fok^KG05913^/C** | 0.453 | 0.231 | 31 | 0.1935 | \|\|\|\|\|\|\| | 0.559 |  |  |  |
| *fok^KG05913^/N** | 0.535 | 0.296 | 31 | 0.3548 | 0.1935 | \|\|\|\|\|\|\| |  |  |  |
| *fok^M110499^/D** | 0.459 | 0.162 | 31 | \|\|\|\|\|\|\| | 0.207 | 0.164 | 2.639 | 0.267 | Negative |
| *fok^M110499^/C** | 0.424 | 0.245 | 19 | 0.2971 | \|\|\|\|\|\|\| | 0.099 |  |  |  |
| *fok^M110499^/N** | 0.482 | 0.161 | 27 | 0.2832 | 0.3509 | \|\|\|\|\|\|\| |  |  |  |

Supplemental Methods

#### Artificial Selection

The locomotor activity of 300 males was recorded over 5 days in a 12:12 LD cycle at 25°C. Using the R library GeneCycle and a custom-made script, we identified rhythmic flies and calculated their ND ratios. In each cycle of selection, we selected 25 males with the most extreme nocturnal or diurnal ND ratios, and crossed them with their (unselected) virgin sisters. The 10 subsequent generations underwent the same selection procedure. In addition, three control groups (CA, CB, CC) were generated from the original population (C_0_) by collecting 25 fertilized females in 3 new bottles. At each selection cycle, the controls underwent the same bottleneck as did the nocturnal (N) and diurnal (D) populations but without any selective pressure. During and following selection, the flies were maintained at 25°C in a 12:12 LD cycle.

Realize heritability was calculated for both the N and D populations from the regression of the cumulative response to selection (as a difference from the original population, C_0_) and the cumulative selection differential base on the data from the 10 cycles of selection (Fig. 1B) [3]. Statistical differences between cycles of selection or between lines were calculated using the Kolmogorov-Smirnov test (KS-test) and the Kruskal-Wallis rank sum test [1,2].

To calculate the correlation between the ND ratios of parents and offspring, we phenotyped 130 virgin males and 130 virgin females from the original copulation (C_0_), as described above, and randomly crossed them. We calculated ND ratios for the progeny of each cross (a male and a virgin female) and correlated this value with parental ND ratios. The correlation between average parent ND ratios and average offspring ND ratios was tested, as were all other possible correlations (i.e., mothers vs. daughters, fathers vs. sons, mothers vs. sons and fathers vs. daughters). For each correlation, we calculated the correlation coefficient and the relative p value.

#### Analysis of fitness-correlated traits

The selected populations were kept in a 12:12LD cycle at 25°C for 5 months (~15 overlapping generation) before testing for differences in viability, fitness and egg to adult developmental time. From the selected N and D populations, as well as the 3 control populations, we collected 40 virgin males and 40 virgin females. For each populations (N, D and pool C), we prepared 10 crosses with 4 males and 4 females. The crosses were maintained with standard sugar food that was changed every 3 days. Dead flies were recorded for each vial, as was the number of adults (progeny) produced and the developmental time (egg to adult).

To analyse the viability of the different populations, we used survival curves. We used the Survival R library to fit Kaplan-Meier curves, which were compared by log-rank tests using χ2 statistics with two degrees of freedom [4]. The progeny produced per vial was recorded, and differences in numbers of progeny per females in the N, C, and D groups werer analysed using ANOVA on log-transformed data. The developmental time (egg to adult) was calculated per vial, per surviving parental female, and ANOVA was used to test for differences among N, C, D population males and females, separately.

#### Locomotor activity

Locomotor activity was recorded as previously described [5], at 25°C. We used the TriKinetics system to record the locomotor activity of 3-4 day-old males maintained initially at in aLD12:12 cycle for 5 days, followed by 7 days in constant conditions (DD conditions) at 25°C. The flies were then trained again in a LD12:12 cycle for 4 days and a 20 min light pulse (saturating white light > 500 lux) was given at ZT15 (3 h after light off) during the last night before releasing the flies in DD conditions for a further 5 days.

The first 5 days in LD conditions were used to calculate ND ratios, the amount of sleep (defined as 5 min of inactivity or longer [6,7]) and the trained morning and evening phases of activity. The phases of morning and evening activity were calculated using cosinor-rhythmometry [8,9]. The amount of sleep during 4 days of a LD cycle was analysed separately for the 12 hours of light (min/12 h) and 12 hours of dark (min/12 h) using nested ANOVA. The first 7 days in DD conditions were used to calculate the free-run period (FRP) and acrophase (φ) using cosinor-rhythmometry [8,9]. Changes in free running period were analysed using ANOVA.

The difference between the phase after the light pulse (φlp) and the reference phase (φref: phase before light pulse) was calculated with a custom-made Excel macro. Circular statistic software (Oriana, Kovach Computing Services, UK) and a Watson-Williams F-test were used to analyse the phase data.

In another set of experiments, we recorded the locomotor activity of the isogenic lines during 5 days in a LD12:12 cycle at 25°C, followed by an 8 h phase delay (realized by a night extension of 8 h) and an additional 8 days in a LD 12:12 cycle at 25°C (Fig. 5B). To measure the light-dependent masking effect, we calculated the ratio (M) between activity during 3 hours after light on and 3 hours before light on (normalized over 24 hours of activity) on the delay day. M ratios were arcsin-transformed for ANOVA analysis. The phase of morning onset (Mon) of activity was recorded for 3 days before the delay and for 4 days after the delay, beginning on the second day. The average Mon of activity before the delay was compared to that for each day after the delay using circular statistic (Oriana, Kovach Computing Services, UK) and a Watson-Williams F-test.

#### Eclosion measurements

Eclosion of adults was recorded automatically as previously described [10], at 25°C in a LD12:12 cycle. TriKinetics DAM2 monitors were placed horizontally on a *Drosophila* eclosion logger adaptor (DELA) [10]. A single fly pupa was placed in modified (shorter) activity tubes just below the infrared sensor of the DAM2 monitor. Because of the strong negative geotaxis of flies, the time a fly needs to cross the infrared sensor after eclosion was minimized. To control for larvae density, groups of 20 males and 20 females were left to lay eggs for 6 hours and the vials were maintained in a LD 12:12 cycle at 25°C. Pupae were collected 2 days pre-eclosion and loaded in the eclosion monitors. Fly gender was scored after eclosion. A Perl script was used to extract eclosion times from the TriKinetics data file (see Suppl. material: eclosion script). Circular statistic software (Oriana, Kovach Computing Services, UK) and a Watson-Williams F-test were used to analyse the eclosion times.

#### Isogenic lines

We generated isogenic lines from the selected populations by crossing three flies from the 8^th^ selection cycle with balancer strains. The nocturnal males had ND ratios of 2.28, 2.22 and 2.10, while the diurnal males had ND ratios of 0.05, 0.05 and 0.07. To isolate chromosome II and III, these males were crossed initially with the double balancer w[1118]/Dp(1;Y)y[+]; CyO/nub[1] b[1] sna[Sco] lt[1] stw[3]; MKRS/TM6B, Tb[1] (FBst0003703, from Dan Lindsley, University of California, San Diego). To isolate chromosome I, the unselected sisters of the selected males were crossed first with the double balancer above and then with Fm7a line (FBst0000785, from Caltech Stock Center). Isogenic lines were generated by mating the progeny of the above crosses.

#### Immunocytochemistry (ICC)

ICC was used to analyse the expression of PER and PDF in the fly brain. For quantification of PDF levels, we used mouse monoclonal anti-PDF and polyclonal rabbit anti-PER antibodies [11,12]. The protocol used for whole-brain staining was previously described [13,14].

We used 3 day-old males and collected and stained their brains after 3 days of training in a LD 12:12 cycle (100Lux LED) at 25°C. Samples were collected every 2 hours beginning at ZT1. After staining, confocal images were obtained using a Leica TCS SPE confocal microscope. The images were visualized and quantified using ImageJ, distribution Fiji. Brightness and contrast were adjusted, but no other manipulations were performed on the images. Samples used for quantification were processed in the same manner during the staining protocol and were scanned with the exact same settings. Nuclear PER signals were quantified by drawing a square-shaped area of 9 pixels (3X3 pixels) in the nucleus of the cell of interest and average pixel intensity was measured at the brightest focal plane. Intensity values from cells of different hemispheres (n=5-10) were background-corrected and averaged for each neuronal group. Quantification of PDF in the neuron terminals was performed according to ref. 12. Our quantification included full projections from the LNv (Fig 7) and we removed all PDF staining outside this area. The resulting images were of the same size and included only PDF projections from the LNv. The background of the image was set to zero and the total intensity of the image was recorded. We quantified between 5 and 12 brains for each time point and genotype. A generalized linear model was fitted to the data that were then analysed using ANOVA to test for the main effects of time and genotype, as well as the time X genotype interaction.

#### RNAseq

RNAseq library preparation and sequencing was carried by Glasgow Polyomics using an IlluminaNextseq500 platform. Two independent libraries (single-end) were generated per time point per line. The data were then processed using Trimmomatic (version 0.32) [15] to remove adapters. The libraries were quality checked using fastQC (version 0.11.2) [16]. The total sequence obtained for each library ranged from 9.7 to 21 Mbp, with a “per base quality score” > 30 phred and a “mean per sequence quality score” > 33 phred. The RNA-seq was aligned to the *Drosophila melanogaster* transcriptome (NCBI_build 5.41) downloaded from the Illumina iGenome website.

We used TopHat (version 2.1.0) [17] for libraries alignment with these options: -i 50 -I 5000 --transcriptome-index NCBI_genes --coverage-search -p 8 Bowtie2Index/genome -G genes.gtf (from NCBI_build5.41) as genome annotation reference. Between 72.8% and 77.7% of sequences were aligned (concordant pair alignment rate), with 1.1-2.5% with multiple alignments and 0.4-1% discordant alignments. Cufflinks and cuffmerge (version 2.2.1) [18,19] were used to quantify the expression of the transcript isoforms of the TopHat-aligned RNA with the following options: –u multi-reads correction, -g genes.gtf (from NCBI_build5.41) as gene annotation reference –b genome.fa (from NCBI_build5.41/Bowtie2Index) as reference genome –M Mask_sequences.fa (including mitochondrial and ribosomal RNA as well polyA, polyC sequences). The expression data were returned in *Fragments Per Kilobase of transcript per Million fragments mapped* (FPKM). To calculate differences of expressions between samples, we used cuffdiff. For cuffmerge, we used –b genome.fa –M Mask_sequences.fa. In addition, we used -u multi-read correction, merged.gtf (the transcript annotation file produced by cuffmerge) and a geometric method for library normalization as described [20]. Samples were compared separately for the 2 time points (2 replicates for each line) or upon merging the time points (4 replicated for each line). This generated a list of differentially expressed genes (p<0.05) per time point and across time points (Table S2). We used the online tool DAVID (<https://david.ncifcrf.gov/>) to identify enriched biological functions in our list of significant differentially expressed genes [21,22].

#### Complementation test

We used a modified version of the quantitative complementation test (QCT) to explore the contribution of several genes to the ND behaviour of the isogenic lines [23,24]. The isogenic lines (N*, D* and C*) were crossed with null mutants, hypomorphic alleles and/or transposable element insertion lines disrupting the normal expression of target genes. The ND ratios of 3-4 day-old F1 males were calculated as described above. For each complementation cross, we used Kolmogorov-Smirnov tests (KS-test) and the Kruskal-Wallis rank sum test (KW-test) with 2 degrees of freedom to test for differences among N*cross, D*cross and C*cross (see Table 4) [1,2]. Positive complementation indicating that genetic variation in the target gene contributes to the ND behaviour. This was confirmed only for the ND D*cross<C*cross<N*cross. The KS-test was significant for the N*cross vs. D*cross contrast and the KW-test was also significant.

**References**

1. Gaddis, G.M., and Gaddis, M.L. (1990). Introduction to biostatistics: Part 5, Statistical inference techniques for hypothesis testing with nonparametric data. Ann. Emerg. Med. *9,* 1054-1059.

2. Nahm, F.S. (2016). Nonparametric statistical tests for the continuous data: the basic concept and the practical use. Korean J. Anesthesiol. *1,* 8-14.

3. Falconer, D.S., and Mackay, T.F.C. (1996). Introduction to Quantitative Genetics Longman).

4. Harrington, D.P., and Fleming, T.R. (1982). A class of rank test procedures for censored survival data. Biometrika *3,* 553-566.

5. Rosato, E., and Kyriacou, C.P. (2006). Analysis of locomotor activity rhythms in Drosophila. Nat. Protoc. *2,* 559-568.

6. Shaw, P.J., Cirelli, C., Greenspan, R.J., and Tononi, G. (2000). Correlates of sleep and waking in Drosophila melanogaster. Science *5459,* 1834-1837.

7. Hendricks, J.C., Finn, S.M., Panckeri, K.A., Chavkin, J., Williams, J.A., Sehgal, A., and Pack, A.I. (2000). Rest in Drosophila is a sleep-like state. Neuron *1,* 129-138.

8. Onyeocha, F.A., and Fuzeau-Braesch, S. (1991). Circadian rhythm changes in toxicity of the insecticide dieldrin on larvae of the migratory locust Locusta migratoria migratorioides. Chronobiol. Int. *2,* 103-109.

9. Nelson, W., Tong, Y.L., Lee, J.K., and Halberg, F. (1979). Methods for cosinor-rhythmometry. Chronobiologia *4,* 305-323.

10. Pegoraro, M., Picot, E., Hansen, C.N., Kyriacou, C.P., Rosato, E., and Tauber, E. (2015). Gene expression associated with early and late chronotypes in *Drosophila melanogaster*. Frontiers in Neurology 100.

11. Stanewsky, R., Frisch, B., Brandes, C., Hamblen-Coyle, M.J., Rosbash, M., and Hall, J.C. (1997). Temporal and spatial expression patterns of transgenes containing increasing amounts of the Drosophila clock gene period and a lacZ reporter: mapping elements of the PER protein involved in circadian cycling. J. Neurosci. *2,* 676-696.

12. Hermann-Luibl, C., Yoshii, T., Senthilan, P.R., Dircksen, H., and Helfrich-Forster, C. (2014). The ion transport peptide is a new functional clock neuropeptide in the fruit fly Drosophila melanogaster. J. Neurosci. *29,* 9522-9536.

13. Hermann, C., Yoshii, T., Dusik, V., and Helfrich-Forster, C. (2012). Neuropeptide F immunoreactive clock neurons modify evening locomotor activity and free-running period in Drosophila melanogaster. J. Comp. Neurol. *5,* 970-987.

14. Hermann, C., Saccon, R., Senthilan, P.R., Domnik, L., Dircksen, H., Yoshii, T., and Helfrich-Forster, C. (2013). The circadian clock network in the brain of different Drosophila species. J. Comp. Neurol. *2,* 367-388.

15. Bolger, A.M., Lohse, M., and Usadel, B. (2014). Trimmomatic: a flexible trimmer for Illumina sequence data. Bioinformatics *15,* 2114-2120.

16. Andrews S. (2010). FastQC: a quality control tool for high throughput sequence data.
Available online at: <http://www.bioinformatics.babraham.ac.uk/projects/fastqc>.

17. Trapnell, C., Pachter, L., and Salzberg, S.L. (2009). TopHat: discovering splice junctions with RNA-Seq. Bioinformatics *9,* 1105-1111.

18. Trapnell, C., Williams, B.A., Pertea, G., Mortazavi, A., Kwan, G., van Baren, M.J., Salzberg, S.L., Wold, B.J., and Pachter, L. (2010). Transcript assembly and quantification by RNA-Seq reveals unannotated transcripts and isoform switching during cell differentiation. Nat. Biotechnol. *5,* 511-515.

19. Trapnell, C., Roberts, A., Goff, L., Pertea, G., Kim, D., Kelley, D.R., Pimentel, H., Salzberg, S.L., Rinn, J.L., and Pachter, L. (2012). Differential gene and transcript expression analysis of RNA-seq experiments with TopHat and Cufflinks. Nat. Protoc. *3,* 562-578.

20. Anders, S., and Huber, W. (2010). Differential expression analysis for sequence count data. Genome Biol. *10,* R106-2010-11-10-r106. Epub 2010 Oct 27.

21. Huang da, W., Sherman, B.T., and Lempicki, R.A. (2009). Bioinformatics enrichment tools: paths toward the comprehensive functional analysis of large gene lists. Nucleic Acids Res. *1,* 1-13.

22. Huang da, W., Sherman, B.T., and Lempicki, R.A. (2009). Systematic and integrative analysis of large gene lists using DAVID bioinformatics resources. Nat. Protoc. *1,* 44-57.

23. Long, A.D., Mullaney, S.L., Mackay, T.F., and Langley, C.H. (1996). Genetic interactions between naturally occurring alleles at quantitative trait loci and mutant alleles at candidate loci affecting bristle number in Drosophila melanogaster. Genetics *4,* 1497-1510.

24. Mackay, T.F., and Fry, J.D. (1996). Polygenic mutation in Drosophila melanogaster: genetic interactions between selection lines and candidate quantitative trait loci. Genetics *2,* 671-688.
